# STAT3 signaling enhances tissue expansion during postimplantation mouse development

**DOI:** 10.1016/j.celrep.2025.115506

**Published:** 2025-04-06

**Authors:** Takuya Azami, Bart Theeuwes, Mai-Linh Nu Ton, William Mansfield, Luke Harland, Masaki Kinoshita, Berthold Gottgens, Jennifer Nichols

**Affiliations:** 1 Cambridge Stem Cell Institute, University of Cambridge, Jeffrey Cheah Biomedical Centre, Puddicombe Way, Cambridge CB2 0AW, UK; 2 Department of Physiology, Development and Neuroscience, University of Cambridge, Tennis Court Road, Cambridge CB2 3EG, UK; 3 Centre for Trophoblast Research, University of Cambridge, Cambridge, UK

## Abstract

Signal transducer and activator of transcription (STAT)3 signaling has been studied extensively using mouse embryonic stem cells. Zygotic deletion of *Stat3* enables embryo implantation, but thereafter, mutants resemble non-affected littermates from the previous day until around mid-gestation. This probably results from the loss of serine-phosphorylated STAT3, the predominant form in early postimplantation embryonic tissues associated with mitochondrial activity. Bulk RNA sequencing of isolated mouse epiblasts confirmed developmental delay transcriptionally. Single-cell RNA sequencing revealed the exclusion of derivatives of *Stat3* null embryonic stem cells exclusively from the erythroid lineage of mid-gestation chimeras. We show that *Stat3* null embryonic stem cells can differentiate into erythroid and hematopoietic lineages *in vitro* but become outcompeted when mixed with wild-type cells. Our results implicate a role for STAT3 in the temporal control of embryonic progression, particularly in tissues requiring rapid cell division, such as postimplantation epiblast and hematopoietic lineages. Interestingly, mutations in *STAT3* are associated with short stature in humans.

## Graphical abstract

**Graphical abstract:**
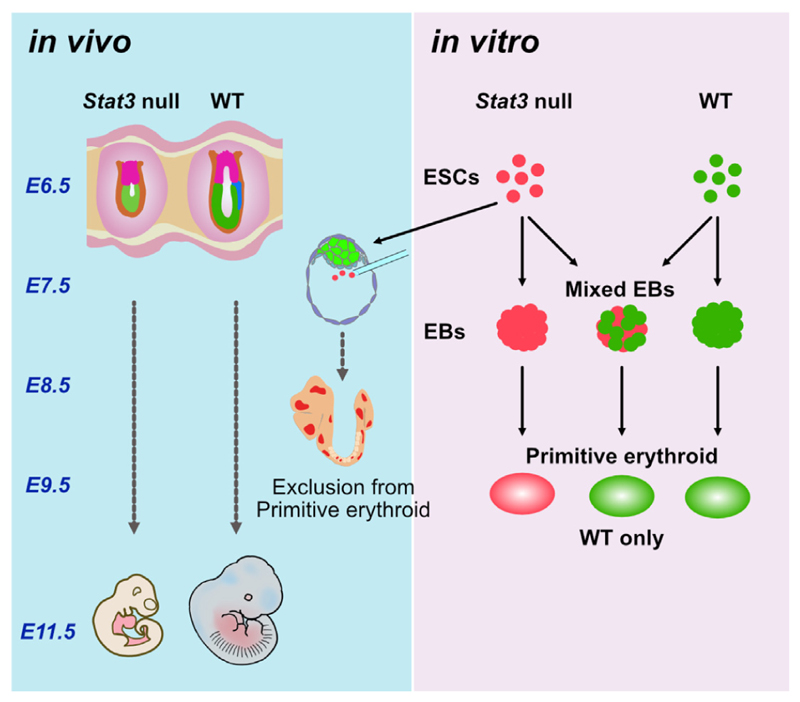

## Introduction

Signal transducer and activator of transcription (STAT)3 is stimulated by the cytokine leukemia inhibitory factor (LIF) in murine embryonic stem cells (ESCs) via activation of Janus-associated kinases (JAKs), inducing phosphorylation of STAT3 at tyrosine 705 (pY705).^1,2^ STAT3 can also be phosphorylated at serine 727 (pS727) by mitogen-activated protein kinases (MAPKs).^3^ Mutation of pY705 ablates ESC self-renewal in standard (serum/LIF) culture conditions, whereas pS727 is apparently not needed in ESCs prior to neuronal differentiation.^3^ A requirement for pS727 was reported for mitochondrial STAT3 transcriptional activity in cell lines, suggestive of a role in metabolic function during differentiation.^4–6^ Zygotic deletion of exons 20–22 of the *Stat3* gene, which contains the SH2 and transactivation domains needed for receptor association and tyrosine phosphodimer formation, produces embryos that can implant in the uterus, but further development appears to be impaired.^7^ STAT3 protein is present in the cytoplasm of oocytes, zygotes, and 2-cell stage embryos, and nuclear-localized pSTAT3(Y705) protein is observed in 4-cell stage embryos.^8^ Deletion of both maternal and zygotically expressed *Stat3* causes early lethality of preimplantation embryos.^9^ In blastocysts, STAT3 regulates cellular metabolism in mitochondria by promoting oxidative phosphorylation and expression of naive pluripotency-associated genes,^10^ but the role of STAT3 at postimplantation stages is largely unknown.

In this study, we outcrossed *Stat3* heterozygous (het) mice^7^ to the robust CD1 genetic background in order to increase litter size and examined postimplantation development. Intriguingly, *Stat3* null embryos could survive beyond gastrulation but exhibited defects in peri-implantation epiblast expansion and the temporal control of embryonic progression and metabolism. Using our recently derived *Stat3* null ESCs,^11^ we explored differentiation and proliferation dynamics *in vitro* alongside wild-type (WT) ESCs derived from the same genetic background. To scrutinize the tissue-forming capacity of embryonic cells lacking STAT3 during development, we generated chimeras by injecting *Stat3* null ESCs into WT CD1 host blastocysts and analyzed their ability to contribute to emerging tissues by means of single-cell chimera sequencing (scChimera-seq) using an updated version of our previously optimized protocol.^12,13^ Although able to colonize most tissues and coordinate developmental stages with the host embryo, *Stat3* null cells were specifically excluded from the primitive blood lineage, consistent with a requirement for STAT3 signaling in tissues associated with rapid proliferation.

## Results

### STAT3 is dispensable for organogenesis but required to sustain developmental pace

**Figure 1:**
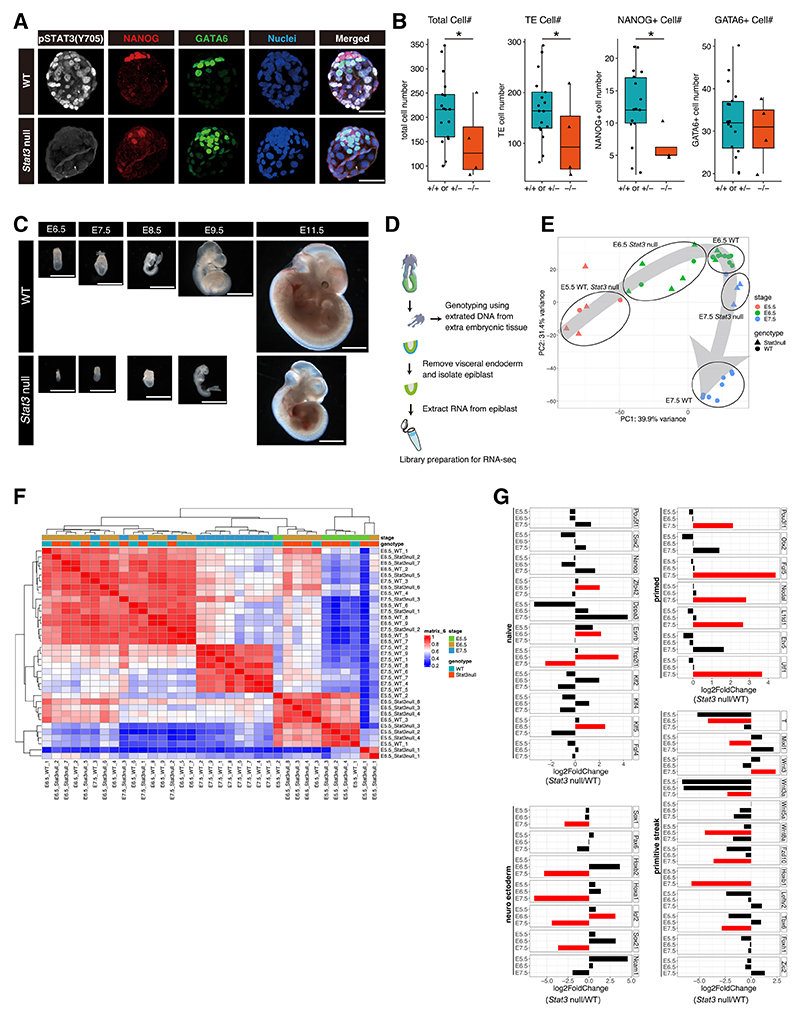
Developmental retardation of *Stat3* null embryos (A) Maximum projection images of immunofluorescence for pSTAT3(Y705) (white), NANOG (red), and GATA6 (green) in E4.5 WT and *Stat3* null embryos. Scale bar: 50 μm. (B) Quantification of cell numbers per embryo from left to right, where each dot represents an embryo: total cells, DAPI+ double negative for NANOG and GATA6 (TE) cells, NANOG+ (epi) cells, and GATA6+ (PrE) cells. Number of WT or het embryos used = 17; number of *Stat3* null = 4; *p < 0.01. (C) Morphology of WT and *Stat3* null embryos dissected at stages from E6.5 to E11.5 by bright-field microscopy. Scale bar: 1 mm. (D) Schematic for dissection of epiblasts isolated from postimplantation embryos and preparation for bulk RNA sequencing. (E) PCA plot of WT and *Stat3* null epiblast cells from E5.5 to E7.5. (F) Heatmap comparison and unsupervised hierarchical clustering of WT and *Stat3* null epiblast cells from E5.5 to E7.5. (G) Log2 fold change comparison (*Stat3* null/WT) for naive, primed, neuroectoderm, and primitive streak genes. Red bar indicates significant differences (adjusted *p* < 0.05).

We recently showed that mouse embryos lacking *Stat3* rapidly lose their inner cell mass (ICM) component when subjected to embryonic diapause,^11^ implicating a requirement for STAT3 signaling for the prolonged survival of epiblasts and primitive endoderm (PrE) in the preimplantation state. Here, we examined embryos not subjected to diapause, generated by inter-crossing *Stat3* het mice on the CD1 genetic background, to determine at which stage developmental deficiencies might arise when *Stat3* null embryos are allowed to implant in a timely manner in the uterus. We first compared peri-implantation embryonic day (E) 4.5 *Stat3* null (*n* = 4) versus WT or het embryos (*n* = 17) using immunofluorescence (IF) for the epiblast (epi) marker NANOG and the PrE marker GATA6 (Figure 1A). Significantly fewer NANOG+ cells were observed in *Stat3* null embryos, whereas the mean numbers of GATA6+ cells did not differ appreciably between genotypes (Figure 1B). A dramatic reduction in the mean number of trophectoderm (TE) cells, identified by the absence of NANOG and GATA6 and the occupation of the outer embryonic layer, in mutant embryos compared with WTs or het mice was observed (Figure 1B). We attribute this phenotype to decreased fibroblast growth factor (FGF) signaling resulting from the small null epiblasts as predicted by previous observations, implying a requirement for FGF4 in trophectoderm proliferation (Figure S1).^10,14,15^

Surprisingly, on the CD1 genetic background, *Stat3* null embryos could be retrieved as late as E11.5 (Table S1) but consistently exhibited a phenotype more closely resembling the morphology expected for the previous day of development compared with their littermates, with no other obvious morphological defects (Figures 1C and S2). This intriguing observation implies that embryos lacking STAT3 are unable to instigate sufficient cell division within the epiblast soon after implantation, when mechanisms for size regulation are normally evoked.^16,17^ To our knowledge, the sustained period of developmental delay we have observed lacking overt signs of abnormalities in organogenesis has not been reported previously.

### STAT3 null epiblasts follow a normal but delayed developmental trajectory

Epiblasts were dissected from CD1 *Stat3* het *inter se* mating at E5.5, E6.5, and E7.5, thus spanning epiblast developmental progression from naive, via the intermediate formative stage,^18^ to primed pluripotency^19^ and the onset of gastrulation. Each embryo was genotyped using extra-embryonic tissue (Figure 1D). From E5.5 to E6.5, the epiblast is basically a homogeneous epithelium poised to respond to asymmetric signals from the extra-embryonic tissues. RNA sequencing (RNA-seq) was therefore performed in bulk on denuded epiblasts to compare transcription profiles between *Stat3* WT and null epiblasts. While E5.5 WT and *Stat3* null samples exhibited very similar transcriptional identities, E6.5 null epiblasts were clearly retarded, with an expression pattern intermediate between WT E5.5 and E6.5 (Figures 1E and 1F). E7.5 null epiblasts grouped more closely with WT E6.5 than E7.5 epiblasts by principal-component analysis (PCA; Figure 1E) and unsupervised hierarchical clustering (Figure 1F). *Stat3* null epiblasts exhibited a downregulation of naive and an upregulation of primed pluripotency genes at E6.5 (Figure 1G), concurrent with preparation for primitive streak formation *in vivo*.^17^ However, mutants failed to induce the neuroectoderm significantly and displayed reduced expression of selected primitive streak genes at E7.5, consistent with the temporal delay of gastrulation (Figure 1G).

### STAT3 activity is directed by pS727 in early postimplantation embryos

**Figure 2:**
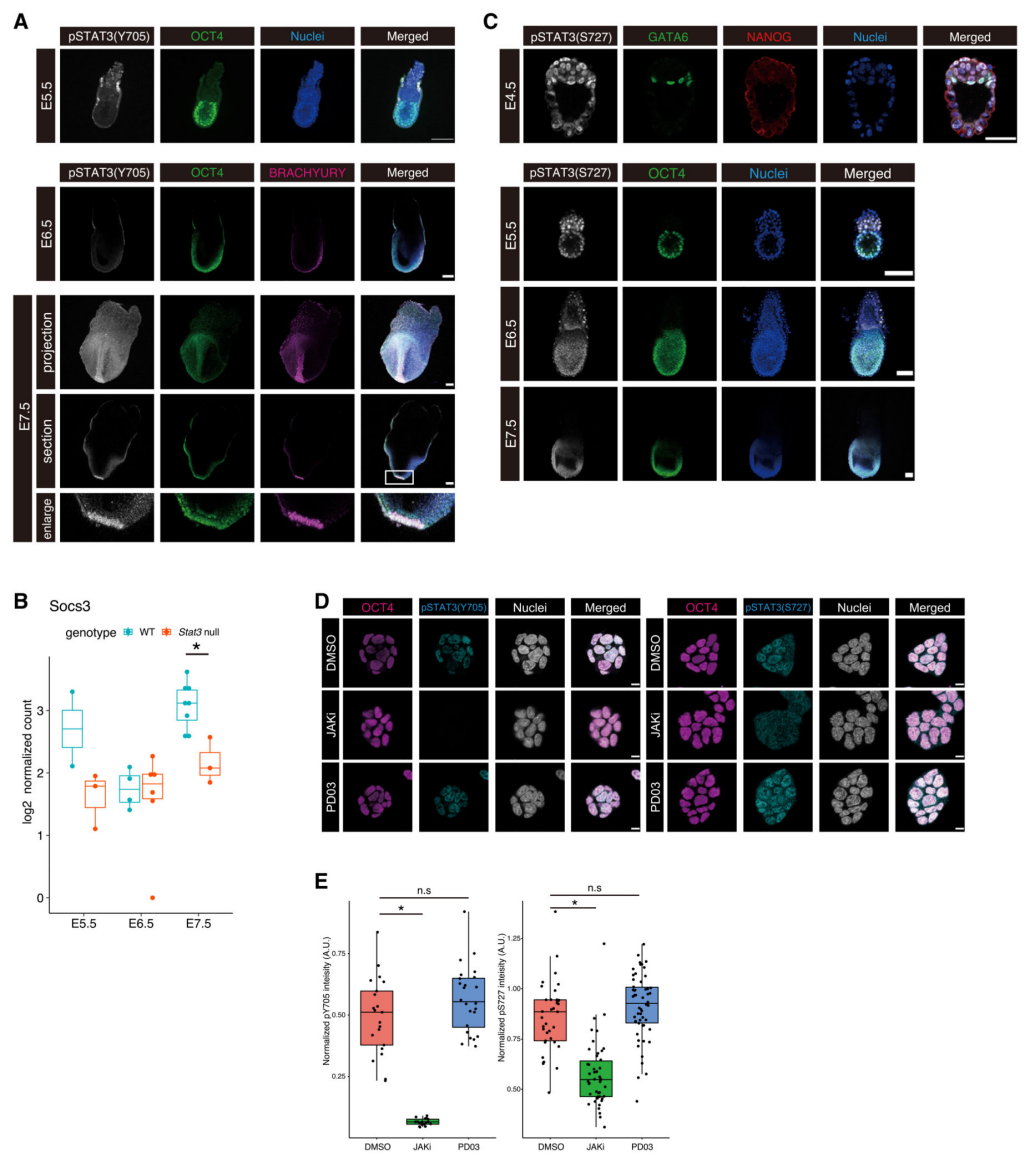
The predominant active form of STAT3 transitions from pY705 to pS727 in WT embryonic lineages during implantation (A) Immunofluorescence of WT embryos for pSTAT3(Y705) and OCT4 at E5.5 and pSTAT3(Y705), OCT4, and BRACHYURY in E6.5 and E7.5 embryos. Scale bar: 100 μm. (B) Log2-normalized expression levels of *Socs3* in WT and *Stat3* null epiblast cells from E5.5 to E7.5. *p < 0.01. (C) Immunofluorescence for pSTAT3(S727), GATA6, and NANOG in E4.5 embryos and pSTAT3(S727) and OCT4 in E5.5, E6.5, and E7.5 embryos. Scale bars: 25 μm for E4.5 and 100 μm for E5.5, E6.5, and E7.5 embryos. (D) Immunofluorescence for pY705 and pS727 STAT3 in mouse ESCs. ESCs were cultured in N2B27 + 2i/L for 24 h and then medium exchanged to N2B27 with DMSO, JAK inhibitor, or PD03 (MEK/ERK inhibitor) and cultured for 2 h. Scale bar: 10 μm. (E) Quantification of pY705 and pS727 STAT3 intensity from immunofluorescence of ESCs as shown in (D). *p < 0.01. n.s, not significant. A.U., arbitrary unit.

Validated antibodies for each of the two STAT3 phosphorylation forms (pY705 and pS727) were used for IF studies on WT CD1 mouse embryos from implantation until gastrulation. As previously shown,^11^ nuclear pY705 STAT3 was detected in peri-implantation embryos at E4.5 (Figure 1A). Apart from the extra-embryonic visceral endoderm, pY705 was barely detectable in WT embryos soon after implantation but re-emerged in the node region of the primitive streak at mid-late gastrulation (Figure 2A). The dramatic reduction of pY705 in postimplantation epiblasts correlates with the low level of the STAT3 target, *Socs3*, revealed in the bulk RNA-seq (Figure 2B). In contrast, pS727 was abundant in the epiblast, extra-embryonic ectoderm, and extra-embryonic endoderm from E4.5 to E6.5, becoming restricted to the visceral endoderm, epiblast, and its derivatives at E7.5 (Figure 2C). These stages coincide with the requirement for elevated mitochondrial function in the embryo to enable rapid cell division for tissue expansion during gastrulation.^20^ To identify potential signaling pathways activated by pS727, we applied candidate inhibitors to WT ESCs to test the phosphorylation reaction *in vitro*. MEK inhibition by PD0325901 (PD03) to block FGF signaling in serum-free medium did not significantly impair the phosphorylation of Y705 or S727 (Figures 2D and 2E), whereas S727 phosphorylation was previously shown to depend upon ERK signaling in serum-containing medium.^3^ Interestingly, JAK inhibition ablated Y705 phosphorylation but only partially dampened pS727, indicating that S727 can be phosphorylated by factors distinct or downstream of JAK signaling or LIF-gp130 receptors (Figures 2D and 2E).

### Self-renewing stem cell lines can be derived from STAT3 null postimplantation epiblasts

**Figure 3:**
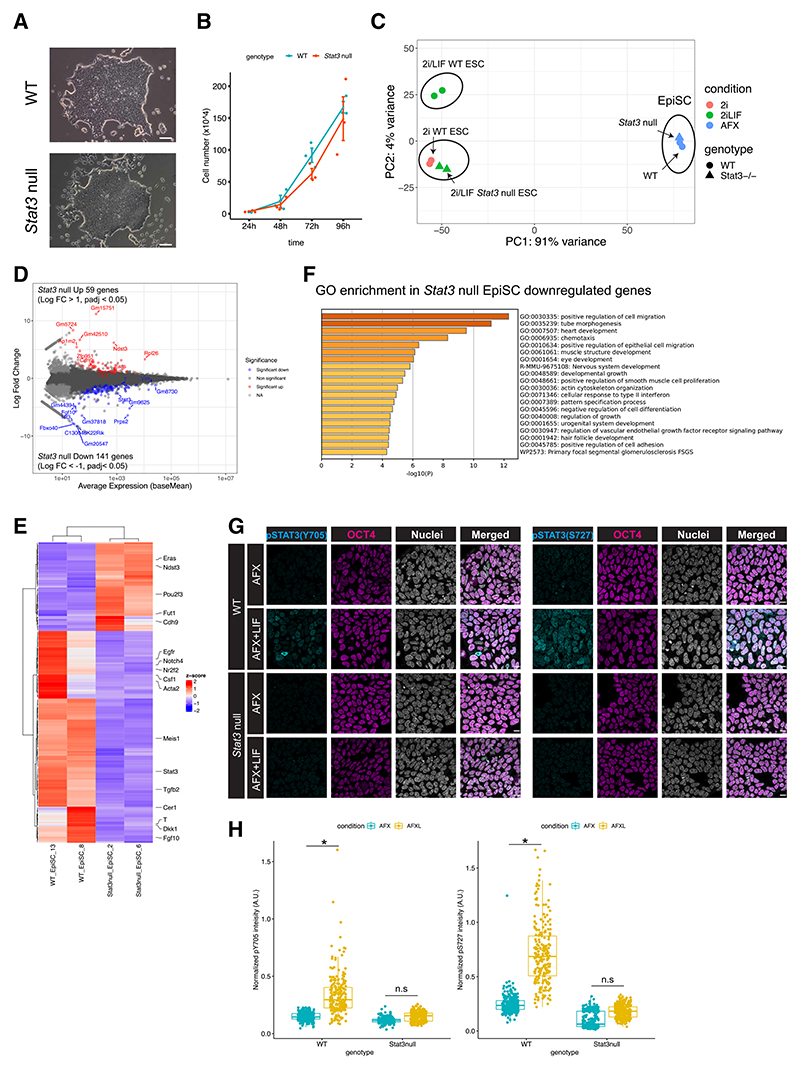
Derivation and analysis of *Stat3* WT and null EpiSCs (A) Representative bright-field images of WT and *Stat3* null EpiSCs derived from epiblasts of E6.5 and E7.5 embryos (Table S2). Scale bar: 100 μm. (B) Proliferation of WT and *Stat3* null EpiSCs. Data are means ± SEM. (C) PCA plot of WT and *Stat3* null ESCs obtained from data in Betto et al.^10^ and EpiSCs from bulk RNA-seq. (D) MA plot of differentially expressed genes (absolute log2 fold change > 1, false discovery rate [FDR] < 0.05) in *Stat3* null EpiSCs related to WT. (E) Heatmap for differentially expressed genes in *Stat3* null EpiSCs compared to WT. (F) Gene Ontology (GO) enrichment analysis for the downregulated genes in *Stat3* null EpiSCs identified in (D). Metascape (https://metascape.org/gp/index.html#/main/step1) was used for the GO analysis. (G) Immunofluorescence for pSTAT3(Y705 and S727) and OCT4 in WT EpiSCs cultured in N2B27+AFX (Activin, FGF2, and XAV) with or without LIF. Scale bar: 10 μm. (H) Quantification of pSTAT3 intensity from immunofluorescence in (G). *p < 0.01. n.s, not significant. A.U., arbitrary unit.

ESCs can be differentiated into formative epiblast-like cells (EpiLCs) when cultured in the presence of activin A and FGF2 for 48 h.^18,21^ *Stat3* null ESCs formed EpiLCs indistinguishable from the WT under this condition (Figure S3). However, the proliferation of *Stat3* null EpiLCs was significantly impaired when differentiated from 2i/LIF culture compared to WT ESCs, suggesting a *Stat3* null *in vitro* defect that could not be overcome during the course of EpiLC differentiation (Figure S3). Interestingly, despite their reduced size compared with WT littermates, epiblasts from *Stat3* null embryos at E6.5 and E7.5 could be isolated and propagated directly as epiblast stem cell (EpiSC) lines^22,23^ with high efficiency (Table S2). Their morphology in culture appeared indistinguishable from that of the WT (Figure 3A), and the growth kinetics were also similar (Figure 3B). Transcriptional profiles of *Stat3* WT and null EpiSCs exhibited close clustering by means of bulk RNA-seq (Figure 3C), implicating a STAT3-independent *in vitro* adaptation to self-renewal during the acquisition of the mid-gastrulation anterior streak identity reported for EpiSCs using original culture conditions, regardless of stage of origin.^24^ Differential expression analysis identified 59 upregulated and 141 downregulated genes in *Stat3* null EpiSCs related to the WT (absolute log2 fold change > 1, adjusted *p* value [*p*adj] < 0.05) (Figures 3D and 3E). Among the significantly downregulated genes in *Stat3* null EpiSCs, the Gene Ontology (GO) terms for cell migration and cell growth were substantially enriched (Figure 3F). Consistent with the small transcriptional changes in *Stat3* null EpiSCs compared to the WT, neither pY705 nor pS727 STAT3 was detected in WT EpiSCs (Figures 3G and 3H), implicating a significant adaptation to culture involving the depletion of the pS727 STAT3, which we had detected in abundance in WT postimplantation epiblasts (Figure 1E). This suggests that although the FGF-ERK pathway phosphorylates S727 STAT3 in ESCs in serum/LIF medium,^3^ pS727 is not induced significantly or maintained by FGF in standard, serum-free EpiSC culture. LIF supplementation was not used for the original EpiSC derivation and culture^22,23^; however, pY705 and pS727 could both be induced by administering LIF to WT EpiSCs (Figures 3G and 3H). We conclude that STAT3 signaling is dispensable for the maintenance of primed pluripotent cells *in vitro*, which is most likely attributable to the relatively slow cell doubling times of around 36 h^25^ characteristic of EpiSCs in culture, compared with pre-primitive streak epiblast cells *in vivo*, which were found to double every 4–5 h.^17^

### *Stat3* null ESCs are specifically excluded from the erythroid lineage in mid-gestation chimeric embryos

*Stat3* null ESCs were previously shown to contribute to all embryonic lineages upon injection into the blastocyst^26^; however, single-cell resolution analyses were not performed to address cell-type-specific contributions at the transcriptional level. Importantly, in that study, the *Stat3* null ESCs were derived on an MF1 outbred background, whereas the host blastocysts were inbred C57BL/6; this disparity in genetic backgrounds creates a competitive environment, which, in this combination, favors the donor cells. This is one motivating factor for the traditional choice of C57BL/6 embryos as hosts for the efficient generation of genetically modified mouse lines. Notably, reduced STAT3 signal transduction was reported in ESCs derived from mice of the FVB, C57BL/6, CBA and non-obese diabetic (NOD) genetic backgrounds compared with the most permissive 129 strain following culture in 2i/LIF.^27^ Therefore, to avoid discrepancies between the donor and host genetic background environments, we injected fluorescently labeled CD1 *Stat3* null ESCs into WT host blastocysts from the same breeding stock to eliminate the risk of encountering confounding inter-strain cell competition effects. We performed scChimera-seq^12,13^ to search for specific differences between *Stat3* null and WT cells within the same embryo and, thereby, identified tissues that may be compromised by the absence of STAT3.

**Figure 4:**
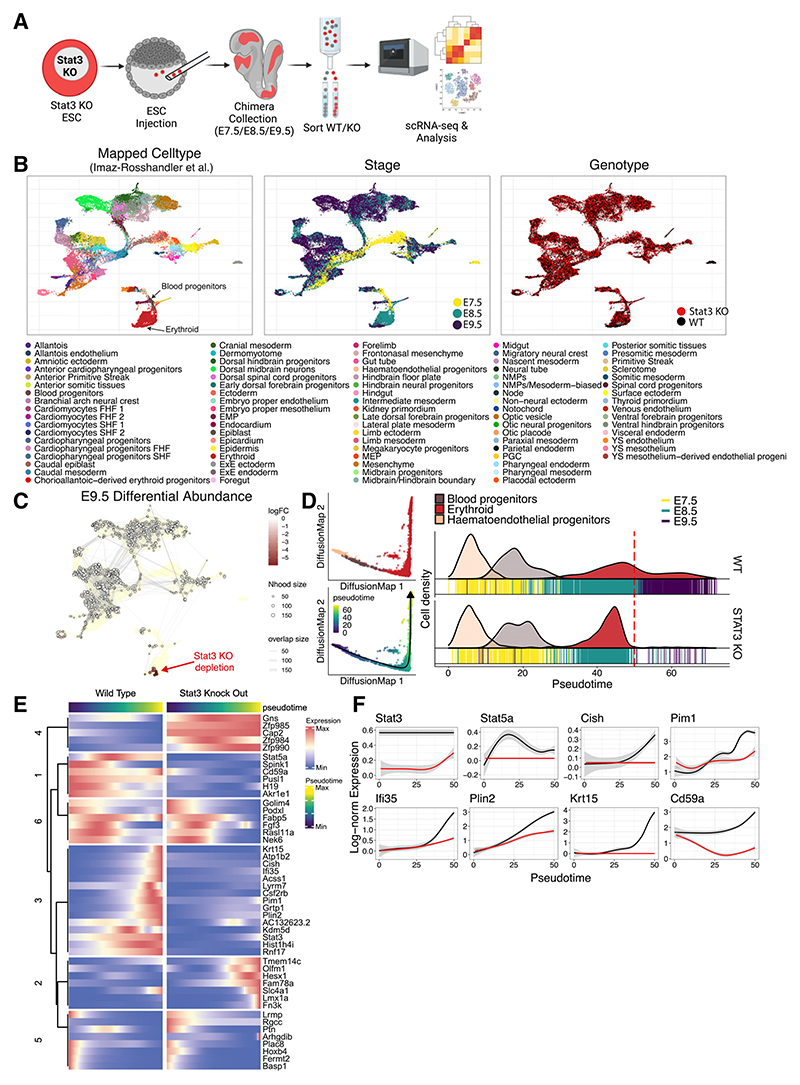
Stat3 null cells are depleted during erythroid lineage differentiation *in vivo* (A) Experimental design for generation of *Stat3* null/WT Chimera-seq experiments. Schematics were created with BioRender.com. (B) Uniform manifold approximation and projection (UMAP) representing transcriptional landscape of single cells from Chimera-seq experiment colored by label-transferred cell type (left), embryonic stage (middle), and genotype (right). (C) Differential abundance between equivalent *Stat3* knockout (KO) and WT cells of E9.5 chimeras. Negative log fold change (logFC) indicates a loss of *Stat3* KO cells in a specific cellular neighborhood. (D) DiffusionMap of hematoendothelial progenitor to erythroid differentiation (left) colored by label-transferred cell type (top) and pseudotime (bottom). Cell density along pseudotime for WT and *Stat3* KO (right). Red dotted line indicates pseudotime cutoff used for downstream analysis. (E) TradeSeq-inferred differential genes between WT and *Stat3* KO along hematoendothelial progenitor to erythroid differentiation. (F) Log-normalized expression of a subset of genes along pseudotime for *Stat3* KO (red) and WT (black).

We demonstrated the high contribution of *Stat3* null ESC derivatives to E9.5 chimeric embryos (Figure S4), thereby validating our experimental approach before embarking upon scChimeraseq. *Stat3* null ESCs were injected into CD1 blastocysts, transferred to pseudopregnant recipient mice, and dissected at E7.5 (*n* = 16), E8.5 (*n* = 6), and E9.5 (*n* = 2). Embryos exhibiting an around 30%–40% contribution of donor cells (Figure S5) were selected for analysis based upon previous optimization of scChimera-seq (Figure 4A).^12,13^ They were dissociated and immediately sorted by flow cytometry into WT versus tdTomato-positive *Stat3* null cells. Cell type annotation of scRNA-seq was performed via label transfer from the previously developed extended atlas of mouse gastrulation and early organogenesis (Figure 4B).^12,28^ *Stat3* null cells exhibited a normal overall contribution to chimeras at the single-cell transcriptional level until E8.5. One notable feature of intact *Stat3* null embryos, revealed by IF for the visceral endoderm markers CER1 and FOXA2, is the reduced intensity and abnormal distribution of the cells comprising the anterior visceral endoderm (AVE), an important signaling center for instructing the formation of anterior structures by the underlying epiblast (Figure S6). In the context of conventional chimeras, the AVE is derived from the host embryo. Support from the WT extra-embryonic tissues accessible to the *Stat3* null cells within chimeras might explain why these injected cells can apparently keep pace developmentally with those of the host embryo in the majority of tissues. However, *Stat3* null cells appeared to be underrepresented in the erythroid lineage at E9.5 (Figures 4B, 4C, and S7). Pseudo-time analysis was performed on erythroid differentiation trajectories in WT and *Stat3* null cells, confirming a significant reduction in contribution to this lineage by *Stat3* null cells in the transition from E8.5 to E9.5 (Figure 4D). Differential gene expression analysis along the erythroid pseudotime revealed dysregulation of canonical *Stat3* target genes, such as *Cish* and *Pim1*, as well as failure to upregulate *Stat5a* expression (Figures 4E, 4F, and S7A). Since *Acss1* (acetyl-coenzyme A [CoA] synthases) and *Lyrm7* (complex III assembly factor) have been shown to function in mitochondrial energy synthesis,^29,30^ their reduced expression in *Stat3* null erythroid cells may explain their restricted contribution to this normally rapidly proliferating lineage (Figures 4E and 4F).

### Differentiation of *Stat3* null ESCs to primitive erythroid cells is impeded when co-cultured with WT cells

**Figure 5:**
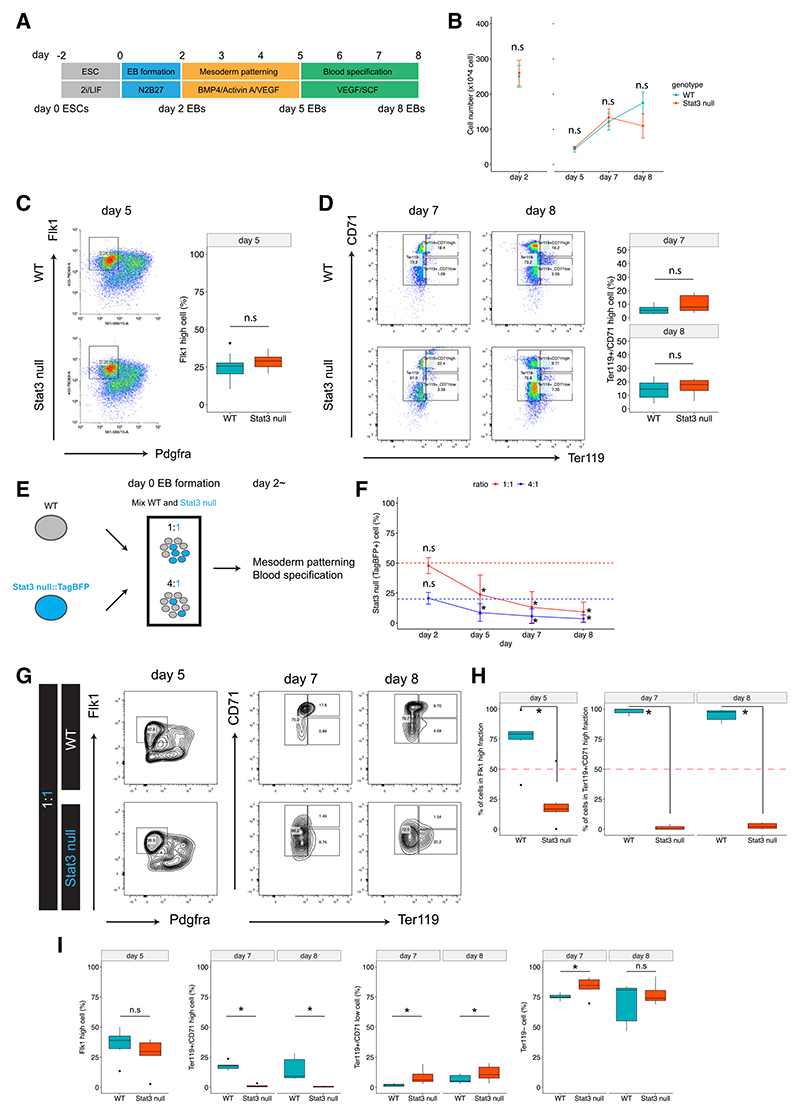
*Stat3* null cells can differentiate into hematovascular mesoderm and blood progenitors *in vitro* but become outcompeted during maturation when mixed with WT cells (A) Schematic for hematovascular mesoderm and erythroid differentiation protocol from mouse ESCs. (B) Cell number in embryoid bodies (EBs) differentiated from WT and *Stat3* null ESCs. EBs were dissociated at day 2 and then reaggregated and cultured for further differentiation without dissociation. n.s, not significant. Data are means ± SEM. (C) Representative flow cytometry analysis of Flk1/Pdgfra expression in day 5 WT and *Stat3* null EBs. Hematovascular mesoderm (Flk1^hi^/Pdgfra^–^) cells are gated in the plot. *n* = 3 (WT) and *n* = 3 (*Stat3* null), two independent differentiations. n.s, not significant. (D) Representative flow cytometry analysis of Ter119/CD71 expression in day 7 and 8 WT and *Stat3* null EBs. Primitive erythroid cells (Ter119^+^/CD71^hi^) are gated in the plot. *n* = 8 (WT) and *n* =8 (*Stat3* null) independent differentiations. n.s, not significant. (E) Schematics for chimeric EB differentiation. WT and TagBFP expressing *Stat3* null ESCs were mixed at 1:1 or 4:1 to make EBs. (F) Percentages of *Stat3* null cells in 1:1 and 4:1 chimeric EBs over the differentiation. Three WT lines and one TagBFP-expressing *Stat3* null ESC line were used, and two independent differentiation experiments were performed and analyzed. *p < 0.05. n.s, not significant. Data are means ± SEM. (G) Representative flow cytometry analysis of Flk1/Pdgfra expression in day 5 and Ter119/CD71 expression in day 7 and day 8 WT and *Stat3* null cells from chimeric EBs, mixing WT and *Stat3* null ESCs at 1:1. (H) Percentages of WT and *Stat3* null cells in the Flk1^hi^ fraction at day 5 and in the Ter119^+^/CD71^hi^ fraction at days 7 and 8. *p < 0.01. (I) Percentages of Flk1^hi^ cells at day 5 and Ter119^+^/CD71^hi^, Ter119^+^/CD71^low^, and Ter119^–^ cells at days 7 and 8 in WT and *Stat3* null cells in chimeric EBs, gated in (G). n.s, not significant. *p < 0.05.

To further investigate the potential of *Stat3* null cells to differentiate into hematopoietic and primitive erythroid lineages, we applied a recently developed *in vitro* differentiation protocol,^31^ which induces yolk-sac-like hematopoietic and endothelial progenitors by supplementing embryoid bodies (EBs) with cytokines (Figure 5A). When cultured separately, the proliferation of *Stat3* null ESCs was equivalent to that of WTs over the differentiation time course until day 8, reflecting the relatively prolonged cell cycle duration at this juncture (Figure 5B). After 5 days of culture, EBs were dissociated and stained for FLK1 and PDGFRA, and the Flk1^hi^/Pdgfra^–^ hematovascular mesoderm population was quantified (Figure 5C). Flow cytometry analysis showed that *Stat3* null ECSs differentiated indistinguishably from WT cells to the hematovascular mesoderm population at day 5. Further differentiation of EBs to hematopoietic progenitors and primitive erythroid lineages was quantified by CD41^+^/c-Kit^+^ and Ter119^+^/CD71^hi^, respectively. *Stat3* null ESCs differentiated to both primitive erythroid and hematopoietic progenitors with equal efficiency compared to WT, suggesting that the development of these lineages by *Stat3* null cells is not inherently impaired (Figures 5D and S8A). This is consistent with our observation that *Stat3* null embryos dissected at E11.5 were not anemic and contained erythroid cells, although they were retarded by 1 day compared with their littermates (Figures 1C and S2). Following our observation of outcompetition of *Stat3* null cells from the erythroid lineage *in vivo* by the WT host embryo, we mixed *Stat3* null ESCs with WTs and performed hematopoietic lineage differentiation on chimeric EBs (Figures 5E and S8B–S8D). The proportions of *Stat3* null to WT cells in mixed EBs maintained initial ratios of 1:1 and 1:4, respectively, but after day 2, *Stat3* null cells became excluded as the EBs embarked upon mesoderm and hematopoietic differentiation (Figure 5F). Despite their reduced contribution to mixed EBs at day 5, *Stat3* null cells were subsequently able to differentiate into the Flk1^hi^/Pdgfra^–^ hematovascular mesoderm but were incapable of forming Ter119^+^/CD71^hi^ primitive erythroid cells (Figures 5G–5I and S9). The failure of *Stat3* null cells to form either of the hematopoietic lineages when competing with surrounding WT cells is consistent with our discovery of the exclusion of *Stat3* null cells from the expanding erythroid lineage in chimeric embryos (Figure 4).

## Discussion

*Stat3* null peri-implantation mouse embryos on the CD1 genetic background exhibited a significant reduction in the number of epiblast cells compared with WT counterparts, whereas the numbers of PrE cells were relatively consistent between both genotypes (Figure 1). This may be explained by the precocious expression of PrE markers, such as *Pdgfra, Sox17*, and *Gata4*, previously observed in *Stat3* null ICMs at the mid-blastocyst stage^10^ that destabilize the lineage-balancing mechanism normally operative within the developing mouse blastocyst.^32^ Previous observations of PrE persistence during diapause when LIF receptor (LIFR) or gp130 is deleted^33^ imply independence of this branch of STAT3 signaling for PrE maintenance, whereas depletion of *Stat3* itself resulted in the loss of the whole ICM.^11^ Somewhat surprisingly, without the interruption of diapause, *Stat3* zygotic null embryos can implant in the uterus and progress through development, presumably by means of the persistent maternally expressed STAT3 already present in the oocyte,^8^ but they appear to lack the regulatory mechanisms that allow compensatory proliferation at the onset of gastrulation that would normally enable size regulation in preparation for organogenesis.^16,17^ The potential consequences of such a defect could be fatal if the number of epiblast cells fails to reach the required threshold, previously shown to be a minimum of 4.^34^ Indeed, this phenomenon may explain the relatively high incidence of empty decidua resulting from het inter-crosses and the sub-Mendelian proportion of *Stat3* null postimplantation embryos recovered (Table S1).

We observed a consistent developmental delay in zygotically deleted *Stat3* mutant postimplantation embryos of approximately 1 day (Figure 1), reminiscent of the reported role for STAT3 signaling in size regulation in later gestation and post-natal growth.^35–37^ However, the original investigation into the role of STAT3 during mouse development exposed a rapid degeneration of *Stat3* null embryos between E6.5 and E7.5, during the period of gastrulation, on the C57BL/6 genetic background.^7^ Since the level of STAT3 signaling differs between mouse strains,^27^ a difference in the strength of signal transduction is most likely the cause of the phenotypic differences observed between CD1 and C57BL/6 genetic backgrounds. *Stat3* null embryos were otherwise apparently normal, suggesting that the transition of epiblasts from naive to primed pluripotency^19^ and subsequent patterning of the fetus can progress independently of STAT3 signaling (Figure 1). Consistent with this, the propagation of EpiSCs derived from postimplantation epiblasts does not require LIF supplementation,^22,23^ but the forced activation of STAT3 or administration of LIF can redirect EpiSCs to an ESC-like state.^38,39^

Our data reveal that STAT3 pY705 protein is negligible in the WT embryo between implantation and organogenesis, whereas persistent phosphorylation during this period was observed for S727 (Figure 2), consistent with a previous report implicating the requirement for pS727 in the exit from pluripotency in the presence of serum *in vitro*.^3^ Since pS727 is considered to be the mitochondrially localized form of STAT3,^4,40^ the reduced growth we observed in *Stat3* null embryos may be a consequence of diminution of mitochondrial function. Bulk RNA-seq also confirmed developmental retardation of approximately 1 day for *Stat3* null epiblasts compared to WT counterparts but following the same trajectory (Figure 1).

*Stat3* null ESCs injected into WT blastocysts contributed to all tissues until mid-gestation, when exclusion from the erythroid lineage was observed (Figure 4). This is the first tissue specified at this stage that requires rapid expansion in order to nurture the growth and survival of the embryo.^41^ In light of the depleted expression of anterior visceral endoderm (AVE) markers CER1 and FOXA2 in the extra-embryonic endoderm exhibited by intact *Stat3* null egg cylinders (Figure S6), we propose that, in the context of a chimera, most *Stat3* null embryonic lineages are at least partially supported by the overlying WT extra-embryonic tissue. Internalization and processing of maternal nutrients are crucial functions of the visceral endoderm, playing an important role in facilitating gastrulation.^42^ The *in vivo* chimera data we present in Figure 4 suggest that *Stat3* null cells may be outcompeted by WTs specifically when required to differentiate in rapidly proliferating lineages. Our *in vitro* EB differentiation study supports this hypothesis: *Stat3* null cells were excluded from the hematovascular mesoderm lineage and further erythroid differentiation when co-cultured with WT cells, whereas *Stat3* null cells were able to differentiate along these lineages normally if cultured alone (Figure 5). STAT3 is known to be required for primitive erythropoiesis via erythropoietin (EPO) in mice and zebrafish.^43–45^ Therefore, *Stat3* null cells may be expected to exhibit defects when differentiated into primitive erythroid cells using EPO. Since *Stat5a* expression was significantly decreased in *Stat3* null cells in chimeric embryos, competition between WT and *Stat3* null cells for EPO signaling would likely exist, and STAT3 might regulate primitive erythropoiesis by fine-tuning the EPO-STAT5 signaling pathway.^43^ The critical aspects of temporal control of embryonic development regulated by the S727 phospho-variant of STAT3 revealed here will inform future studies on erythroid differentiation in the absence of STAT3 and the development of protocols to investigate the interaction of embryonic and extra-embryonic tissues during gastrulation.

Mutations in STAT3 signaling are implicated as the cause of various defects in humans, affecting a range of organs and processes. Non-lethal effects include short stature, autoimmunity, and erythropoiesis defects,^46^ diagnosed at birth and persisting to adulthood.^47–49^ Many are the result of point mutations, often causing gain-of-function effects; the likely consequences of these include disruption to STAT3 function.^35^ We propose that these human post-natal growth defects may begin during early postimplantation development, resulting from the challenges imposed upon the epiblast to undergo rapid proliferation in preparation for gastrulation. Our discovery that mouse embryos lacking STAT3 can exhibit prolonged developmental delay beyond organogenesis supports this hypothesis and provides a novel system with which to dissect mechanisms associated with the regulation of proportional organ expansion and overall size regulation.

## Limitations of the study

(1) We report a generally consistent developmental delay of around 1 day in mouse embryos lacking STAT3 compared to their WT and het littermates, persisting from soon after implantation until E11.5. Our RNA-seq analysis confirmed the observed developmental retardation at the transcriptional level, but this phenotype made it difficult to pinpoint differences in gene expression attributable specifically to the loss of STAT3. To confound our attempts further, the well-known target genes of STAT3 were identified by the investigation of pSTAT3(Y705) in cell lines, which, we discovered by our IF studies of embryos, is absent from the postimplantation epiblast.
(2) For mouse embryos, even within a litter, there is always some variation in the stages between conceptuses, although never by as much as 1 day. Differences between the developmental paces of males versus females may contribute to intra-litter variability. Furthermore, natural intra- and inter-litter deviation is likely to contribute to variability between samples.
(3) Loss of *Stat3* in developing embryos is not compatible with birth or post-natal development, which may be attributable to late developmental defects or compression from the larger neighboring littermates. As previously shown and noted in the discussion, implanting mouse embryos require at least 4 epiblast cells in order to proceed with development. The non-Mendelian, reduced provision of *Stat3* null embryos often encountered (see Table S1) meant that some of our samples of *Stat3* null embryos were quite small compared with the excess of het/WT embryos.
(4) Stem cell lines derived from embryos provide a useful resource for many studies since they can be expanded indefinitely. However, during the derivation and expansion process, some phenotypic and molecular changes occur, so they may exhibit several distinct properties from their founder tissues. Most importantly, in the context of this study, their speed of cell division generally decreases.

## Resource Availability

### Lead contact

Further information and requests for resources and reagents should be directed to and will be fulfilled by the lead contact, Jennifer Nichols (jenny.nichols@ed.ac.uk).

### Materials availability

WT and STAT3 null ESC and EpiSC lines are available upon request.

## Star ✶ Methods

### Key Resources Table

**Table:** 

REAGENT or RESOURCE	SOURCE	IDENTIFIER
Antibodies		
Mouse monoclonal anti-Oct3/4 (C-10)	Santa Cruz	Cat#SC-5279; RRID:AB_628051
Goat polyclonal anti-Brachyury	R&D systems	Cat#AF2085; RRID:AB_2200235
Rat monoclonal anti-Nanog	eBioscience	Cat#14-5761-80; RRID:AB_763613
Rat monoclonal anti-OCT3/4	eBioscience	Cat#14-5841-82; RRID: AB_914301
Rat monoclonal anti-SOX2	eBioscience	Cat# 14-9811-82; RRID:AB_11219471
Rabbit anti-phospho-(Tyr705) STAT3	Cell Signaling Technology	Cat#9145; RRID:AB_2491009
Rabbit anti-phospho-(Ser727) STAT3	Cell Signaling Technology	Cat#34911; RRID:AB_2737598
Goat anti-GATA6	R&D systems	Cat#AF1700; RRID:AB_2108901
Goat anti-CER1	R&D systems	Cat#AF1075; RRID:AB_2077228
Rabbit anti-FOXA2	abcam	Cat# ab108422; RRID:AB_11157157
BV786 conjugated anti-Flk1	BD Biosciences	Cat# 740938; RRID: AB_2740568
PE conjugated anti-Pdgfra	eBiosicence	Cat# 12-1401-81; RRID:AB_657615
PE conjugated anti-CD71	eBiosicence	Cat# 12-0711-82; RRID:AB_465740
FITC conjugated anti-Ter119	eBiosicence	Cat# 11-5921-82; RRID:AB_465311
PE-Cy7 conjugated anti-CD41	eBiosicence	Cat# 25-0411-82; RRID:AB_1234970
APC conjugated anti-*c*-Kit	eBiosicence	Cat# 17-1171-82; RRID:AB_469430
Donkey anti-rat IgG Alexa 488	Thermo Fisher Scientific	Cat# A-21208; RRID:AB_2535794
Donkey anti-mouse IgG Alexa 488	Thermo Fisher Scientific	Cat# A-21202; RRID:AB_141607
Donkey anti-rabbit IgG Alexa 555	Thermo Fisher Scientific	Cat# A-31572; RRID:AB_162543
Donkey anti-goat IgG Alexa 64	Thermo Fisher Scientific	Cat# A-21447; RRID:AB_2535864
Donkey anti-mouse IgG Alexa 647	Thermo Fisher Scientific	Cat# A-31571; RRID:AB_162542
Chemicals, peptides, and recombinant proteins		
Recombinant mouse LIF	Qkine	Qk018
Recombinant human activin A	Qkine	Qk001
Recombinant zebrafish Fgf2	Qkine	Qk002
Recombinant human BMP4	Qkine	Qk038
Recombinant human SCF protein	Qkine	Qk078
Recombinant human VEGF 165 protein	Qkine	Qk048
N2 Supplement	In house	N/A
B27 Supplement	Thermo Fisher Scientific	17504044
Neurobasal	Thermo Fisher Scientific	11540566
DMEM/F12	Thermo Fisher Scientific	21103049
Human Plasma Fibronectin	Merck Millipore	FC010
Prcine Gelatin	Sigma Aldrich	G-1890
Accutase	Biolegend	423201
TrypLE Express Enzyme	Thermo Fisher Scientific	12605010
M2 medium	Sigma-Aldrich	M-7167
KSOM medium	Merck Millipore	MR-101-D
PD0325901	abcr	AB 253775
CHIR99021	abcr	AB 253776
Y27632	TOCRIS	1254
JAK inhibitor I	Calbiochem	420099
XAV939	TOCRIS	3748
Lipofectamine2000	Thermo Fisher Scientific	11668027
Opti-MEM	Thermo Fisher Scientific	31985062
Anti-Adherence Rinsing Solution	STEMCELL TECHNOLOGIES	07010
HEPES	Thermo Fisher Scientific	15630056
L-Glutamine	Thermo Fisher Scientific	25030024
β-mercaptoethanol	Thermo Fisher Scientific	31350-010
Hoechst 33342	Thermo Fisher Scientific	62249
IMDM	Thermo Fisher Scientific	12440053
Ham’s F-12 Nutrient Mix	Thermo Fisher Scientific	11765054
Bovine Albumin Fraction V (7.5% solution)	Thermo Fisher Scientific	15260037
1-Thioglycerol	Sigma Aldrich	M6145
L-Ascorbic Acid	Sigma Aldrich	A4403
Critical commercial assays		
Ribo-Zero rRNA Removal Kit	Illumina	MRZH11124
PicoPure RNA Isolation kit	Thermo Fisher Scientific	KIT0214
NEBNext Ultra II DNA Library Prep Kit for Illumina	New England Biolabs	E7645S
SMARTerR Stranded Total RNA-Seq Kit v2 - Pico InputMammalian	Takara Clontech	634412
RNeasy Mini Kit	QIAGEN	74104
Deposited data		
Bulk RNA-seq	This paper	GSE260590
scChimera-seq	This paper	GSE260590
RNA-seq	Betto et al.^10^	GSE133926
Experimental models: Cell lines		
Mouse/CD-1 ESC, screened negative for mycoplasma	Kraunsoe et al.^11^	N/A
Mouse/CD-1 Stat3^−/ −^ESC, negative for mycoplasma	Kraunsoe et al.^11^	N/A
Mouse/CD-1 EpiSC, negative for mycoplasma, authenticated via marker IF	This paper	N/A
Mouse/CD-1 Stat3^−/ −^EpiSC, negative for mycoplasma, authenticated via marker IF	This paper	N/A
Experimental models: Organisms/strains		
Mouse CD-1 adult males and females	WT-Gurdon Institute, University of Cambridge	N/A
Mouse/Stat3^−/ −^ adult males and females, maintained under UK Home Office License P76777883, intercrossed by natural mating to generate embryos	Takeda et al.^7^	N/A
Pregnant females were culled by cervical dislocation by a trained personal license holder and embryos dissected at E4.5 to E11.5. Sex ND.	Dissected from uteri at required stage after appearance of copulation plug	N/A
Oligonucleotides		
Genotyping primers	See Table S3	N/A
Recombinant DNA		
pPBCAG-H2B-tdTomato-IP	This paper	N/A
pPBCAG-TagBFP-IP	This paper	N/A
Software and algorithms		
HISAT2(v2.2.1)	Johns Hopkins University, Center for Computational Biology	https://daehwankimlab.github.io/hisat2/howto/
StringTie (v2.1.4)	Johns Hopkins University, Center for Computational Biology	https://ccb.jhu.edu/software/stringtie/
ComplexHeatmap (version 2.1.0)	N/A	https://doi.org/10.18129/B9.bioc.ComplexHeatmap
biomaRt (version2.50.3)	N/A	https://doi.org/10.18129/B9.bioc.biomaRt
DESeq2 (v1.30.1)	Love et al.^50^	https://bioconductor.org/packages/release/bioc/html/DESeq2.html
CellRanger (v6.0.1)	10x Genomics Inc.	N/A
R (v4.2.2)	N/A	https://www.R-project.org/
scran (1.26)	Bioconductor	https://bioconductor.org/packages/release/bioc/html/scran.html
scuttle (1.8)	Bioconductor	https://bioconductor.org/packages/release/bioc/html/scuttle.html
scds (v1.14)	Bioconductor	https://www.bioconductor.org/packages/release/bioc/html/scds.html
batchelor (v1.14)	Bioconductor	https://bioconductor.org/packages/release/bioc/html/batchelor.html
BiocNeighbors (v1.8.2)	Bioconductor	https://www.bioconductor.org/packages/release/bioc/html/BiocNeighbors.html
irlba (v2.3.5.1)	Cran	https://cran.r-project.org/web/packages/irlba/index.html
miloR (1.6)	Bioconductor	https://www.bioconductor.org/packages/release/bioc/html/miloR.html
edgeR (v3.40)	Bioconductor	https://bioconductor.org/packages/release/bioc/html/edgeR.html
destiny (3.12)	Bioconductor	https://www.bioconductor.org/packages/release/bioc/html/destiny.html
slingshot (v2.6)	Bioconductor	https://www.bioconductor.org/packages/release/bioc/html/slingshot.html
tradeSeq (v1.12)	Bioconductor	https://www.bioconductor.org/packages/release/bioc/html/tradeSeq.html
Metascape	Zhou et al.^51^	https://metascape.org/gp/index.html#/main/step1
Fiji (2.16.0)	Fiji community, ImageJ	https://fiji.sc/
CellProfiler (4.2.1)	Broad Institute	https://cellprofiler.org/
FlowJo (10.10.0)	BD Biosciences	https://www.flowjo.com/
Code		https://github.com/BartTheeuwes/Stat3_code
Other		
Accession code	Gene Expression Omnibus data	GSE260590

### Experimental Model and Study Participant Details

#### Mice, husbandry and embryos

Experiments were performed in accordance with EU guidelines for the care and use of laboratory animals and under the authority of appropriate UK governmental legislation. Use of animals in this project was approved by the Animal Welfare and Ethical Review Body for the University of Cambridge, covered by relevant Home Office licences. Mice were maintained on a lighting regime of 12:12 h light:dark with food and water supplied *ad libitum*. STAT3 mice heterozygous for replacement of exons 20–22 with Neomycin resistance^7^ were backcrossed extensively to CD1 mice. Embryos were generated from *Stat3*^+/−^ *inter se* natural mating. Detection of a copulation plug in the morning after mating indicated embryonic day (E) 0.5. Embryos were isolated in M2 medium (Sigma).

#### Genotyping

Mice were genotyped by PCR using ear biopsies collected within 4 weeks of birth and genomic DNA was extracted using Extract-N-Amp tissue prep kit (Sigma-Aldrich). Embryos were genotyped using either immune-reactivity to antibody raised against STAT3 pY705 or pS727 in the case of those imaged for confocal analysis, or PCR analysis of trophectoderm lysate for ESC derivation or surplus extraembryonic tissue for postimplantation embryos. Amplification was carried out on around 5 μL of lysate for 35 cycles (following 95°C hot start for 10 min) of 94°C, 15 s; 60°C, 12 s; 72°C, 60 s, with a final extension at 72°C for 10 min. Reaction products were resolved by agarose gel electrophoresis. Primers used for genotyping PCR are listed in Table S3.

### Method Details

#### Histology

Post-gastrulation embryos were fixed in 4% PFA overnight, and serially dehydrated in increasing concentrations of ethanol, rinsed twice in 100% ethanol and embedded in paraffin. Microtome sections of 8 μm thickness were examined histologically via haematoxylin and eosin (H&E) staining.

#### Cell cultures

Cell lines are listed in the key resources table. Cell lines were cultured without antibiotics in humidified incubators at 37°C in 7% CO_2_. Reduced oxygen (5%) was used for mouse EpiSCs. Cell lines tested negative for mycoplasma by periodic PCR screening.

#### Derivation and culture of ESC and EpiSC

Morulae were collected from het females 2.5 days after mating by het males and used for ESC derivation as described previously^52^ by culture to the blastocyst stage in KSOM supplemented with 2i, consisting of 1 μM PD0325901 and 3 μM CHIR99021, transfer of ICMs isolated by immunosurgery^53^ to 4-well plates containing 2i in N2B27 medium, one per well. WT and *Stat3* null ESCs were expanded and maintained in N2B27 supplemented with 2i or 2i/LIF on gelatin-coated plates at 37°C in 7% CO_2_ and passaged by enzymatic disaggregation every 2–3 days. To examine phosphorylation of STAT3 in ESCs, WT cells were cultured in N2B27 with 2i/LIF for 24 h then N2B27 supplemented with DMSO, 1 μM PD0325901, or 0.5 μM JAK inhibitor I (JAKi, Calbiochem) for 2 h. For EpiSC derivation, epiblasts were manually isolated from E6.5 or E7.5 embryos obtained from *Stat3* het inter-cross by removal of extraembryonic ectoderm and visceral endoderm by means of flame-pulled Pasteur pipettes of appropriate diameter. Genotyping was performed using genomic DNA extracted from extraembryonic tissue as described above. Isolated epiblasts were plated on fibronectin-coated plates in N2B27 medium supplemented with AFX, consisting of 12 ng/mL FGF2, 20 ng/mL Activin A and 2 μM XAV929. EpiSCs were maintained in N2B27 supplemented with AFX in fibronectin coated-plates at 37°C in 5% O_2_ and passaged every 3–4 days. To examine phosphorylation of STAT3 in EpiSCs, WT and *Stat3* null EpiSCs were cultured with 10 ng/mL LIF in N2B27 +AFX medium for 1 h.

#### Transfection

To generate stable tdTomato and TagBFP-expressing *Stat3* null ESC lines, cells were plated in 24-well plates the day before transfection. 2 μg of plasmid DNA and 2 μL of Lipofectamine2000 (Thermo Fischer Scientific #11668019) were incubated in 25 μL Opti-MEM (gibco) for 5 min, mixed and incubated for 20 min at room temperature. The mixture was added to cells and cultured for 3 h, then exchanged for fresh medium. After 24 h, cells were passaged to gelatin-coated 6-well plates and puromycin (1 μg/mL) selection performed. Expanded colonies were picked manually and cell lines uniformly expressing tdTomato and TagBFP selected by flow cytometry.

#### Blood progenitor differentiation

WT and *Stat3* null ESCs were differentiated into mesoderm and blood progenitors as previously described,^31^ with modifications to the protocol as follows: at day 0, the cells were plated at 5x10^5^ cell to 60 mm dish in serum-free differentiation (SF-D) medium,^54^ and allowed to aggregate as embryoid bodies (EBs). After 48h, EBs were collected and dissociated into single cells by TrypLE Express Enzyme (Gibco). Single cells were plated at 1x10^5^ cell/well in 12-well plate in SF-D media containing recombinant human bone morphogenetic protein 4 (rhBMP4, 10 ng/mL, Qkine), Activin A (5 ng/mL, Qkine), and recombinant human vascular endothelial growth factor (rhVEGF, 5 ng/mL, Qkine). At day 5, EBs were washed and split 1:2 in SF-D media containing rhVEGF (5 ng/mL, Qkine) and stem cell factor (SCF, 50 ng/mL, Qkine). From day 5, EBs were cultured in 12-well plate coated with anti-adherence rinsing solution (STEM CELL TECHNOLOGIES).

#### Flow cytometry

EBs were collected and dissociated into single cells in TrypLE Express Enzyme (Gibco). Cells were washed three times in FACS buffer (2% FCS in PBS), stained with antibodies diluted in FACS buffer for 30 min on ice. Samples were analyzed using BD LSR Fortessa or CytoFLEX S (Beckman Coulter). Flow cytometry data was analyzed using FlowJo software (BD Biosciences). Antibodies used for flow cytometry analysis are listed in the Key resources table.

#### Immunofluorescence

Embryos were fixed in 4% paraformaldehyde (PFA) for 30 min at room temperature (RT), followed by washing in 0.5% polyvinylpyrrolidone in PBS. Embryos were permeabilized in 0.5% Triton X-100 in PBS for 15 min and blocked with 2% donkey serum, 2.5% BSA, and 0.1% Tween 20 in PBS for 1 h at RT. For phosphorylated-STAT3 staining, permeabilization was performed in absolute methanol for 10 min at −20°C. Primary antibodies were diluted in blocking solution and incubated overnight at 4°C. After washing in 0.1% Tween 20 in PBS, embryos were incubated with Alexa Fluor-conjugated secondary antibodies (Thermo) for 1–3 h at RT. Nuclear staining was carried with Hoechst33342 (Thermo). Primary and secondary antibodies used are listed in the Key resources table.

For IF of ESCs and EpiSCs, cells were cultured on fibronectin coated ibidi-treated 8-well chamber slides (ibidi). Cells were fixed in 4% PFA for 15 min at RT, followed by washing in PBS. Cells were permeabilized in absolute methanol for 10 min at −20°C, then washed in PBS. Blocking reaction was carried out with 2% donkey serum, 2.5% BSA, and 0.1% Tween 20 in PBS for 1 h at RT. Primary antibodies were diluted in blocking solution and incubated overnight at 4°C. After washing in 0.1% Tween 20 in PBS, cells were incubated with Alexa Fluor-conjugated secondary antibodies (Thermo) for 1 h at RT. Nuclear staining was carried with Hoechst33342 (Thermo).

#### Library preparation for bulk RNA-sequencing

For low-input RNA-sequencing, embryos from E5.5 to E7.5 were dissected from *Stat3* heterozygous inter-crosses, and extraembryonic ectoderm and visceral endoderm removed manually using a flame-pulled Pasteur pipette of appropriate diameter. Extraembryonic ectoderm was used for genotyping. RNA was isolated from single epiblasts with Picopure RNA-isolation kit (Thermo). For library construction, SMARTerR Stranded Total RNA-seq kit v2-Pico InputMammalian (Takara Clontech) was used. For bulk RNA-sequencing for EpiSCs, total RNA was isolated using RNeasy Mini Kit (QIAGEN). Ribo-Zero rRNA Removal kit (Illumina) was used for rRNA removal and NEBNext Ultra II DNA Library Prep Kit for Illumina was used for the library construction.

#### Blastocyst injection of *Stat3* null ESCs

tdTomato-expressing Stat3 null ESCs were dissociated with Accutase and resuspended in 20 mM HEPES containing N2B27 medium. Around 10 cells were injected into each E3.5 CD1 blastocyst, incubated in N2B27 at 37°C in 7% CO_2_ for ~1 h to allow re-expansion, then transferred to pseudo pregnant female mice generated by mating to vasectomised males 2.5 days previously. Dissected embryos were imaged using a Leica stereo microscope. For sectioning, embryos were fixed in 4% PFA for overnight, which was replaced with 20% sucrose/PBS and incubated overnight at 4°C then embedded in OCT compound and sectioned at 8 μm thickness. Sections were imaged using Zeiss apotome microscope.

#### Chimera-seq analysis

Sample preparation for Chimera-seq was performed as previously reported.^12^ Briefly, chimeric embryos were collected at E7.5 (16 embryos), E8.5 (6 embryos), and E9.5 (2 embryos). Embryos were dissociated into single cells by TrypLE Express Enzyme, then tdTomato^+^(*Stat3* null) and tdTomato^–^(WT) cells were sorted using a BD Influx sorter for subsequent 10x scRNA-seq library preparation and sequencing on an Illumina HiSeq 4000 platform.

#### scRNA-seq pre-processing

Raw sequencing files were mapped to the mm10 reference genome and counted with the GRCm38.p5 annotation including the Tomato-Td gene using CellRanger count with chemistry = SC3Pv3. All downstream processing and analysis were performed in R. High quality cells were retained using the following thresholds: log10(number of reads) > 4 & < 5, number of genes > 2e3 & < 1e4, percentage mitochondrial RNA <5%, percentage ribosomal RNA <30%. Count normalization was performed by first calculating size factors using the computeSumFactors function from scran,^55^ where cells were pre-clustered using scran’s quickCluster with method = igraph, minimum and maximum sizes of 100 and 3000 cells per cluster, respectively, followed by logNormCounts from scuttle.^56^ Doublet calling was performed with the scds^57^ package using the hybrid approach and doublet score >1 as threshold. Accidental sorting of Stat3 KO cells into the WT samples was corrected by annotating whether a cell from a WT sample contained any read mapping to the Tomato-Td gene, and those cells were excluded from downstream differential abundance and differential expression testing.

#### Label transfer from extended transcriptional atlas of mouse gastrulation and early organogenesis

Label transfer was performed for cell-type assignment by mapping to the E7.5 – E9.5 stages of the extended gastrulation atlas.^28^ First, the reference atlas was subset to a maximum of 15k cells per embryonic stage and all cells for the ‘mixed-gastrulation’ stage. Mapping was then performed for every sample separately using the batchelor package,^58^ each sample is referred to as the ‘query’. Joint normalisation was performed by first running MultiBatchNorm for the reference atlas with samples as batch, followed by co-sineNorm over the logcounts of both the reference atlas and the query. For downstream analysis, non-informative and noisy genes were removed, these include the genes which names start with Rik, Mt, Rps, Rpl, or Gm, end with Rik, hemoglobin genes, imprinted genes, Xist and Tsix, Tomato-Td, and Y chromosome genes. This was followed by selecting the top 2,500 highly variable genes in the reference atlas using modelGeneVar (Scran) with samples as block, which are used for downstream analysis. Dimensionality reduction was performed using MultiBatchPCA for the concatenated query and reference atlas objects, with the ‘atlas’ and ‘query’ as batch, and d = 40. Batch correction was performed in multiple rounds using ReducedMNN, where first the atlas was corrected within each embryonic stage with samples ordered from largest to smallest, followed by atlas correction between embryonic stages with embryonic stages ordered from latest to earliest. This was followed by batch correction between the reference atlas and the query. Nearest neighbors (NN) of every query cell in the reference atlas were identified using queryKNN from the BiocNeighbors package with k = 25. Cell type label transfer was then performed by taking the mode of the cell types of the NN, ties were broken by taking the cell-type of the reference atlas cell closest to the query.

#### Dimensionality reduction

Dimensionality reduction was performed by first detecting the top variable genes in the same way as described in the ‘label transfer from extended transcriptional atlas of mouse gastrulation and early organogenesis‘ section. Next, the number of genes and reads were regressed out as covariates in the log-normalised count matrix. Next, PCs were calculated using the prcomp_irlba function from the irlba package, with *n* = 40. Batch correction was performed as described for the reference atlas above, correcting both within and between embryonic stages. Uniform Manifold Approximation and Projection (UMAP) was performed using Scater’s run-UMAP function on batch-corrected PCs, with n_neighbors = 30 and min_dist = 0.3.

### Quantification and Statistical Analysis

#### Imaging and image analysis for ESCs and EpiSCs

Images of ESCs and EpiSCs were acquired with LSM980 (Zeiss) confocal microscope and processed with ImageJ. Segmentation of nucleus and quantification of pSTAT3 levels were performed using CellProfiler 4.2.1.^59^ Mean intensity value of pSTAT3 in each nuclear section was normalized by mean intensity of Hoechst channel. Statistical analyses were performed using the nonparametric Mann–Whitney *U*-test on R environment.

#### Bulk RNA-seq analysis

Reads were aligned to the mouse (GRCm38/mm10) reference genome using HISAT2(v2.2.1). StringTie (v2.1.4) was used for gene counts. R environment and Bioconductor were used for all RNA-seq analysis. Normalization and differential expression analysis were carried out with DESeq2 package (v1.30.1). Transcripts with absolute log2 fold change >1 and the *p*-value adjusted (padj) < 0.05 considered as differentially expressed gene. Regularized log transform (rlog) normalized values were used for principal component analysis (PCA) and heatmap. GO-term enrichment analysis was performed with Metascape.^51^ WT and *Stat3* null ESC bulk RNA-seq data was obtained from.^10^

#### Chimera-seq - Differential abundance testing

Differential abundance testing was performed for each embryonic stage separately using the MiloR package.^60^ First, embryonic stage specific dimensionality reduction and batch correction was performed as described above, except for the between embryonic stage batch correction step. Next, Milo was performed on embryonic stage specific batch corrected PCs by first running buildGraph with k = 30 followed by makeNhoods with k = 30 and prop = 0.05. Cells per neighborhood were counted using countCells and differential abundance testing was performed using testNhoods with design = ~ embryo pool + genotype, where each embryo pool contains one sample for both the Stat3 KO and the WT condition that were collected as chimeric embryos and separated by the presence or absence of tdTomato expression by fluorescence-activated cell sorting.

#### Chimera-seq - Cell type differential gene expression testing

Differential gene expression testing was performed on the subset of genes after filtering out non-informative and noisy genes as described in the ‘label transfer from extended transcriptional atlas of mouse gastrulation and early organogenesis‘ section. Next, cells were pseudobulked by their label transferred cell type, original sample, and genotype, where only those conditions with more than 15 cells were retained. Differential genes were then detected using Scran’s pseudoBulkDGE function, with label = the label transferred cell types, the design the same as for the testNhoods function in ‘Differential abundance testing’, and the genotype as the condition. The significance thresholds were set at FDR<0.05 and log2 fold change >1.

#### Chimera-seq - Erythroid trajectory

Cells labeled as ‘Hematoendothelial progenitors’, ‘Blood progenitors’, or ‘erythroid differentiation trajectory’ were used for inference of the erythroid trajectory. Gene filtering and top 1,000 highly variable gene selection was performed as described in the ‘label transfer from extended transcriptional atlas of mouse gastrulation and early organogenesis‘ section. PCs were calculated using Scater’s runPCA with ncomponents = 5 and PCs were used to generate the diffusionmap using the DiffusionMap function from the destiny package.^61^ Pseudotime was estimated using slingshot^62^ with PCs as input. For differential expression testing along pseudotime, only cells with a pseudotime value below 50 were used to have comparable densities for both genotypes. Genes were filtered as described above, with the additional filter of genes having to be detected in at least 10% of cells in either of the genotypes. Differential expression testing was performed using the TradeSeq package.^63^ First, a negative binomial generalized additive model was fitted for every gene using fitGAM with condition = ‘genotype’, U = ‘embryo pool’, pseudotime = ‘slingshot pseudotime’, nknots = 6, and cellWeights set to ‘1’ for every cell as all cells belong to the same trajectory. Next, differential genes were detected using conditionTest with l2fc = 1. Multiple-comparison correction was done using p.adjust with method = ‘fdr’ and genes with corrected *p*-values of <0.05 were labeled as significant.

### Additional Resources

None.

## Supplementary Material

**Supplemental Information**

Supplemental information can be found online at https://doi.org/10.1016/j.celrep.2025.115506.

Supplementary Material

## Data Availability

Bulk RNA-seq and scChimera-seq data have been deposited in the Gene Expression Omnibus (GEO) with the accession codes GEO: GSE260590 and are publicly available as of the date of publication. The code used to perform these analyses is available at https://github.com/BartTheeuwes/Stat3_code. Any additional information required to reanalyze the data reported in this work paper is available from the lead contact upon request.

## Highlights

- STAT3 null embryos exhibit developmental delay persisting beyond mid-gestation
- Serine 727 phospho-STAT3 predominates during postimplantation epiblast expansion
- STAT3 null ESC derivatives are excluded from blood lineages in chimeric embryos
- WT outcompetes STAT3 null ESCs in primitive erythroid differentiation *in vitro*

## In brief

Azami et al. reveal that STAT3 deletion depletes epiblast cells at implantation, causing a persistent developmental delay of around a day. STAT3 null embryonic stem cells contribute to all embryonic tissues in chimeras except blood. Mixing with wild-type cells for *in vitro* erythroid differentiation causes the outcompetition of STAT3 null cells.
