# Supplementary material for: STAT3 signaling enhances tissue expansion during postimplantation mouse development
Source: Cell Rep. Author manuscript; Available in PMC 2025 Dec 7. (PMC7618442; doi:10.1016/j.celrep.2025.115506)
Supplement: Supplementary Material [file EMS211403-supplement-Supplementary_Material.pdf]

Cell Reports, Volume *44*

Supplemental information

STAT3 signaling enhances tissue expansion during postimplantation mouse development

Takuya Azami, Bart Theeuwes, Mai-Linh Nu Ton, William Mansfield, Luke Harland, Masaki Kinoshita, Berthold Gottgens, and Jennifer Nichols

Supplementary Figure 1

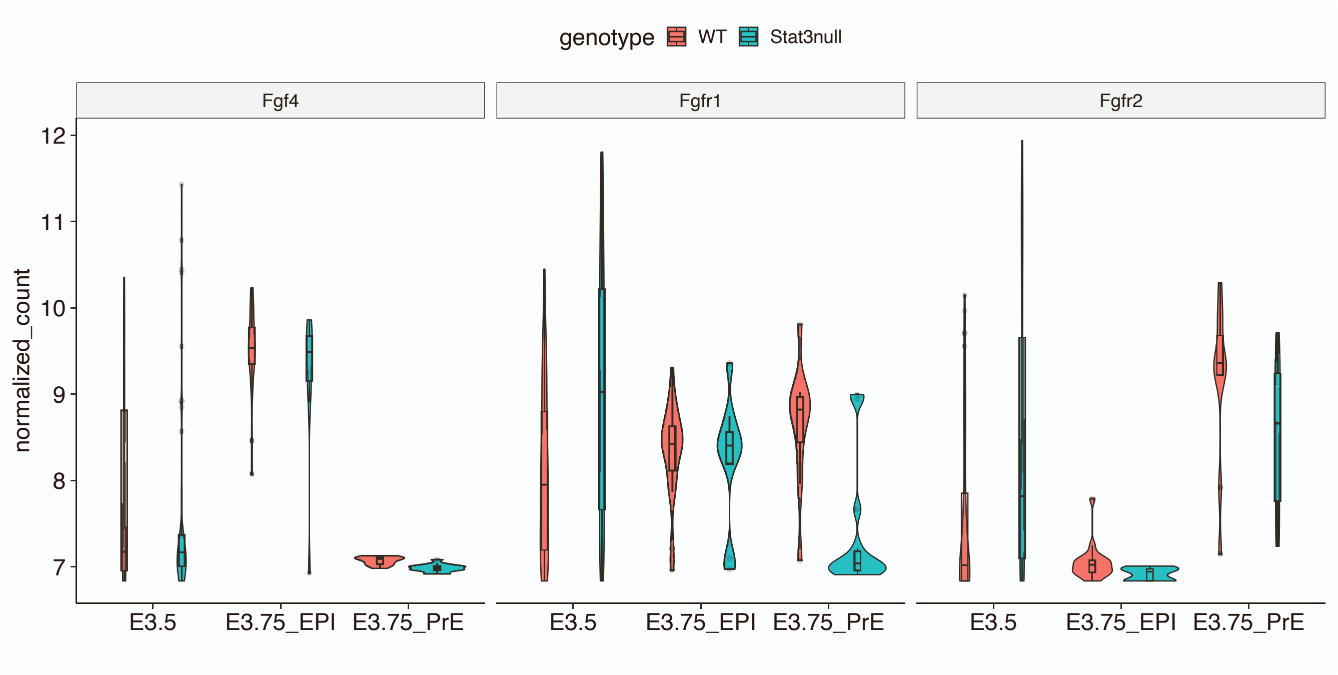

Supplementary Figure 1. Expression of Fgf signaling related genes in WT and *Stat3* null pre-implantation embryos.

Normalized expression levels of *Fgf4, Fgfr1,* and *Fgfr2* in WT and *Stat3* null E3.5 ICM and E3.75 epiblast (EPI) cells and primitive endoderm (PrE) cells from scRNA-seq analysis data (Betto et al., *Nat Genet*, 2021).

Supplementary Figure 2

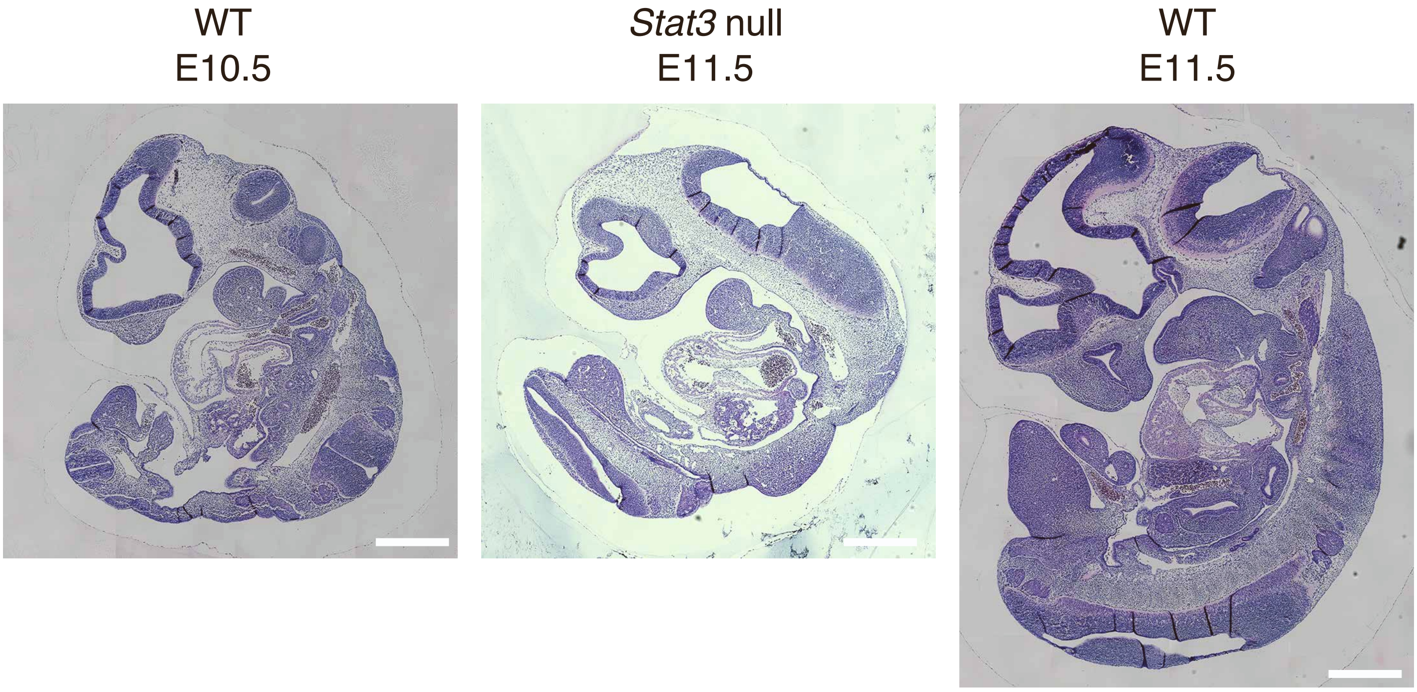

Supplementary Figure 2. Histological sections for WT and *Stat3* null embryos.

Hematoxylin and eosin staining for E10.5/11.5 WT and E11.5 *Stat3* null embryos. Scale bar = 500 μm.

Supplementary Figure 3

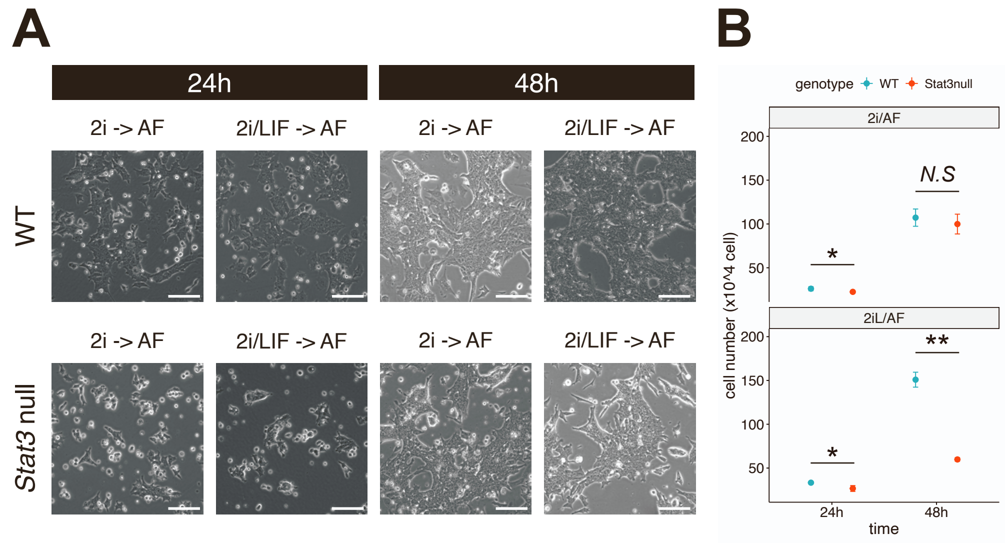

Supplementary Figure 3. EpiLCs differentiation from WT and *Stat3* null ESCs.

(A) Representative bright field images of EpiLCs differentiated from 2i or 2i/LIF cultured WT and *Stat3* null ESCs in N2B27 supplemented with Activin A and FGF2 at 24 h and 48 h. Scale bar = 100 μm. (B) Cell numbers of WT and *Stat3* null EpiLCs differentiated for 24 h and 48 h. * $p < 0.05$. ** $p < 0.01$. n.s, not significant.

Supplementary Figure 4

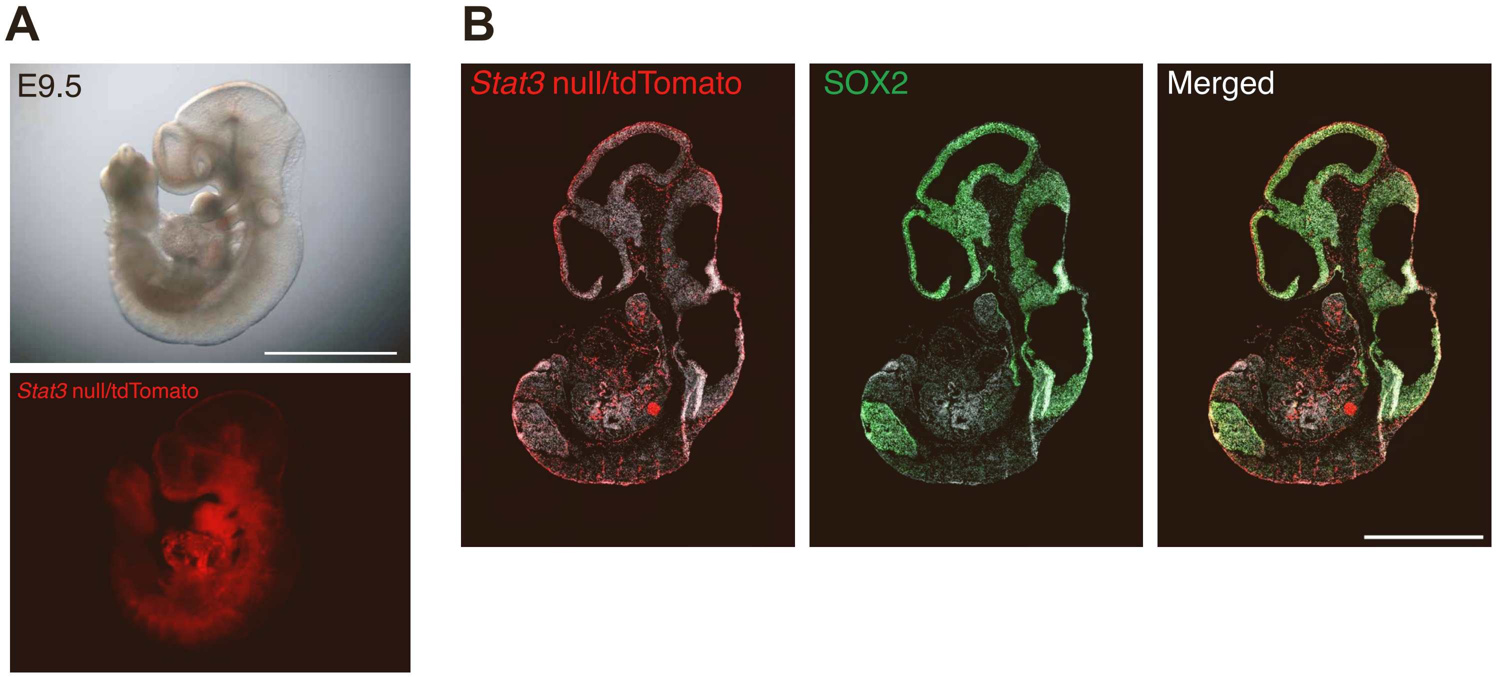

Supplementary Figure 4. Contribution of *Stat3* null cells in chimeric embryos.

(A) Bright field (top) and tdTomato fluorescent (bottom) images of chimeric embryos. tdTomato-expressing *Stat3* null ESCs were injected host blastocyst and chimeric embryos were collected at E9.5. Scale bar = 50 μm. (B) Immunofluorescence for SOX2 in chimeric embryos. Note tdTomato-positive *Stat3* null cells contributed to multiple tissues in the embryo, including SOX2-positive neural lineages in the brain. Scale bar = 50 μm.

Supplementary Figure 5

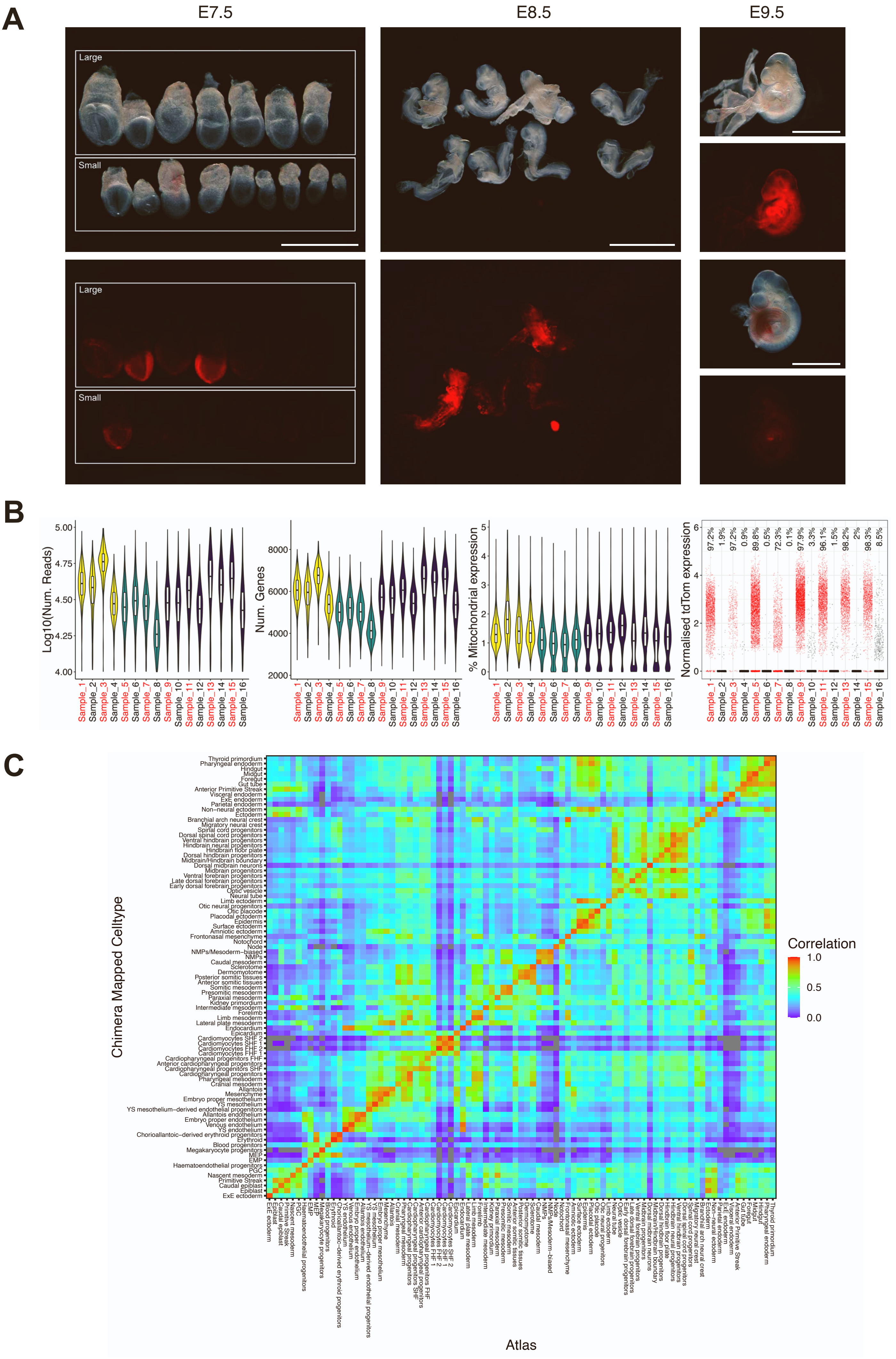

Supplementary Figure 5. Chimera embryos used for single-cell Chimera-seq and quality control of single-cell chimera-seq.

(A) Bright field and tdTomato fluorescent images for E7.5, E8.5, and E9.5 chimeric embryos used for single-cell Chimera-seq. For E7.5, embryos were classified by their size (large and small). 7 large and 9 small E7.5 embryos were pooled and processed for scRNA-seq. For E8.5, 3 or 4 embryos were pooled and processed for scRNA-seq. For E9.5, two embryos were processed individually for scRNA-seq. Scale bar = 50 μm. (B) Quality of chimaera-seq cells per sample after filtering, showing number of reads, number of genes, percentage of mitochondrial expression, and normalised tdTomato expression including percentage of cells that are non-zero. *Stat3* KO and WT samples are in red and black, respectively. E7.5 – yellow, E8.5 – green and E9.5 – purple. (C) Heatmap showing Pearson correlation of expression of cell type marker genes between cell types of the extended transcriptional atlas of mouse gastrulation and early organogenesis and label transferred cell types of the *Stat3* KO chimera.

Supplementary Figure 6

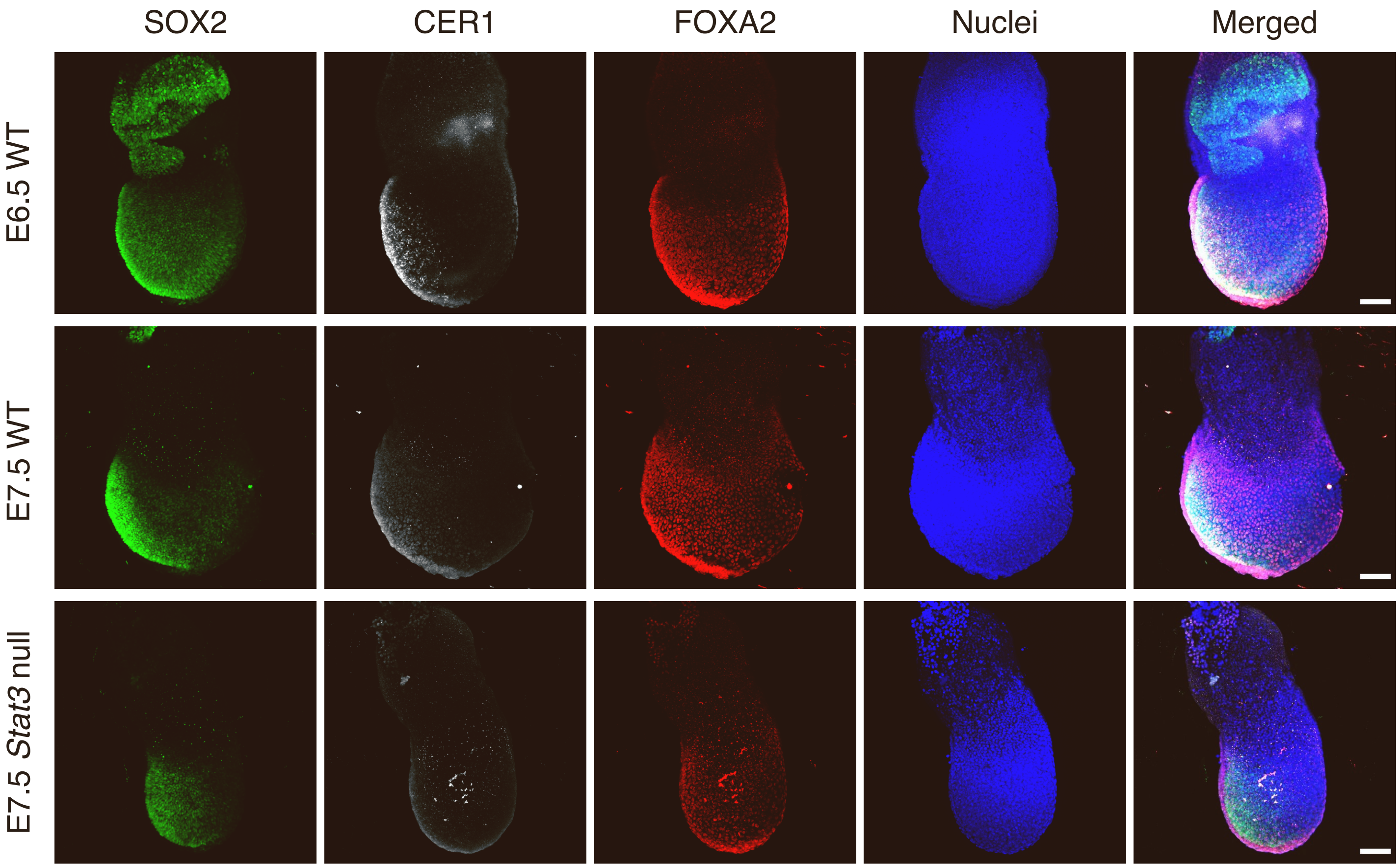

Supplementary Figure 6. Extra-embryonic endoderm development in *Stat3* null embryos.

Immunofluorescence for SOX2, CER1, and FOXA2 in E6.5/7.5 WT and E7.5 *Stat3* null embryos. E6.5 WT (n = 5), E7.5 WT (n = 6), and E7.5 *Stat3* null (n = 3) were examined. Scale bar = 50 μm.

Supplementary Figure 7

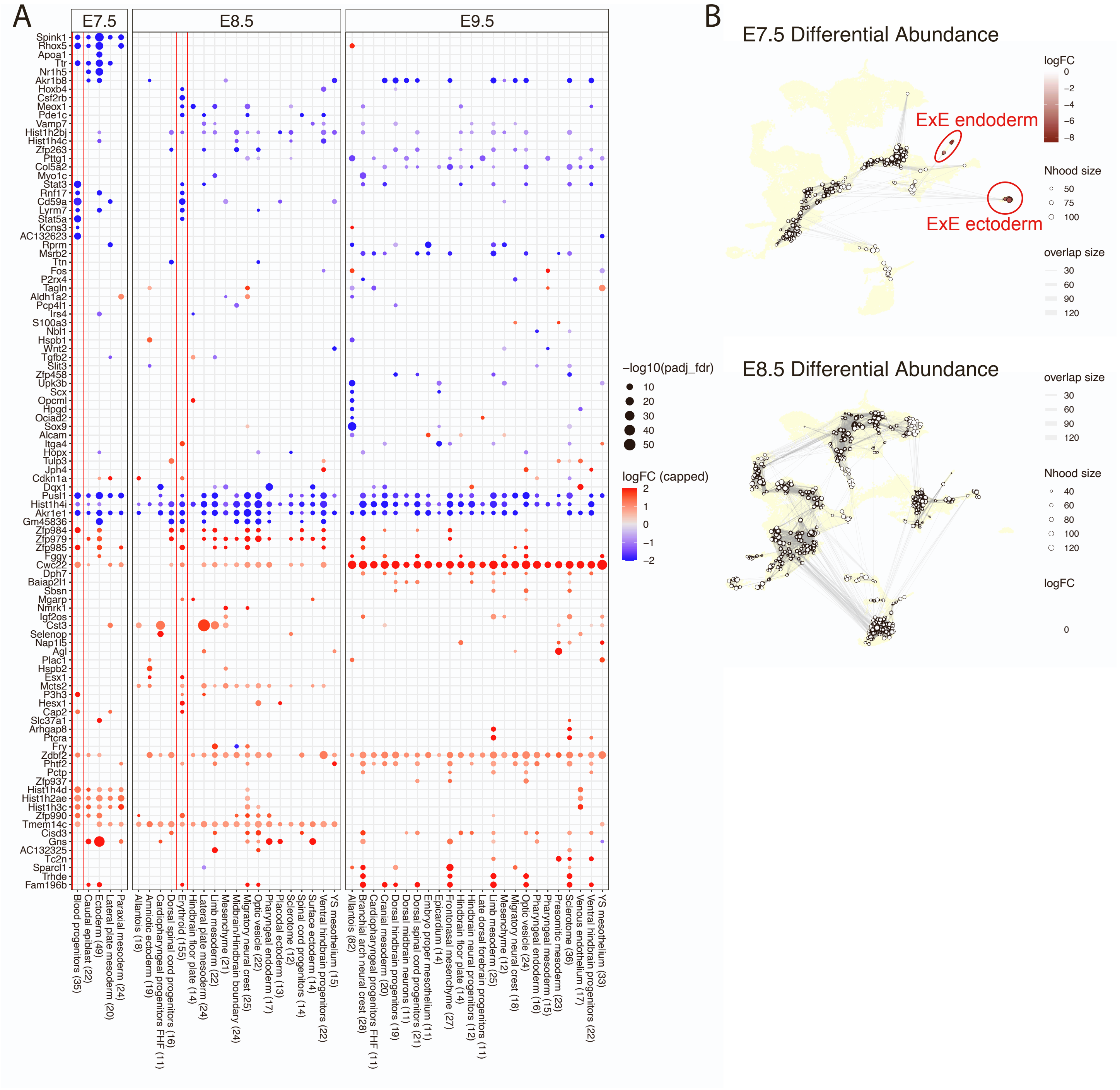

Supplementary Figure 7. Differential expression and abundance of *Stat3* null and WT cells in E7.5, E8.5, and E9.5 chimeras.

(A) Differential expression between *Stat3* null and WT cells for label transferred cell types at each embryonic stage. Only cell types with more than 10 differential genes and genes differentially expressed in more than 1 cell types are shown. Positive values represent genes that are more highly expressed in *Stat3* null. (B) Differential abundance testing between *Stat3* null and WT cells from E7.5 and E8.5 chimera-seq data. Negative logFC indicates a loss of *Stat3* null cells in a specific cellular neighbourhood.

Supplementary Figure 8

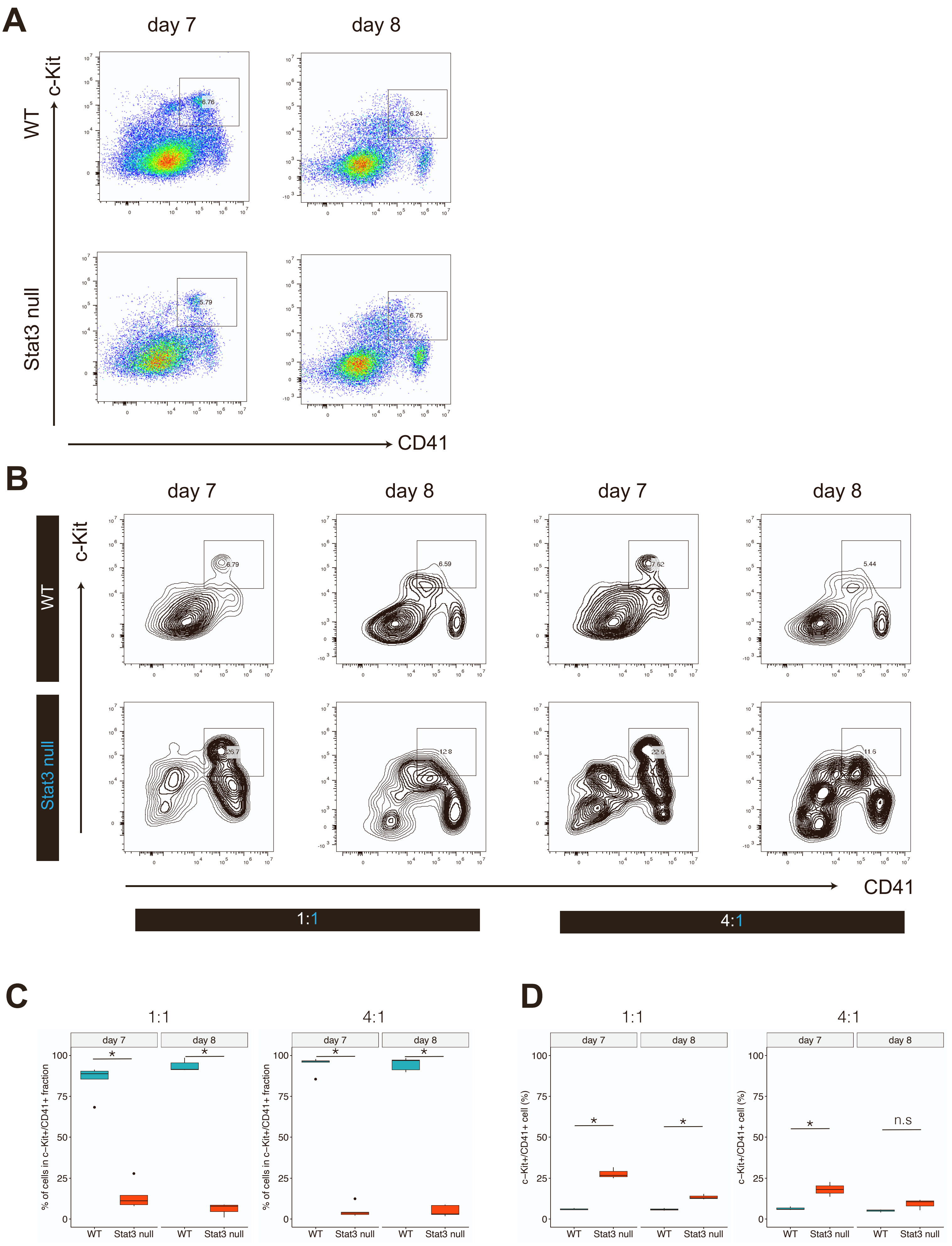

Supplementary Figure 8. Hematopoietic progenitor differentiation from *Stat3* null ESC.

(A) Representative flow cytometry analysis of CD41/c-Kit expression in day 7 and 8 WT and *Stat3* null EBs. $CD41^+$/c-Kit^+ cells are gated in the plot. n = 8 (WT) and n = 8 (*Stat3* null) independent differentiation. (B) Representative flow cytometry analysis of CD41/c-Kit expression at day 7 and day 8 in WT and *Stat3* null cells in chimeric EBs, mixing WT and *Stat3* null ESCs at 1:1 and 4:1. n = 3 independent differentiation. (C) Percentages of WT and *Stat3* null cells in the c-Kit^+/$CD41^+$ fraction at day 7 and day 8. * $p < 0.05$. (D) Percentages of c-Kit^+/$CD41^+$ fraction at day 7 and day 8 in WT and *Stat3* null cells in chimeric EBs, gated in (B). n.s, not significant. * $p < 0.05$.

Supplementary Figure 9

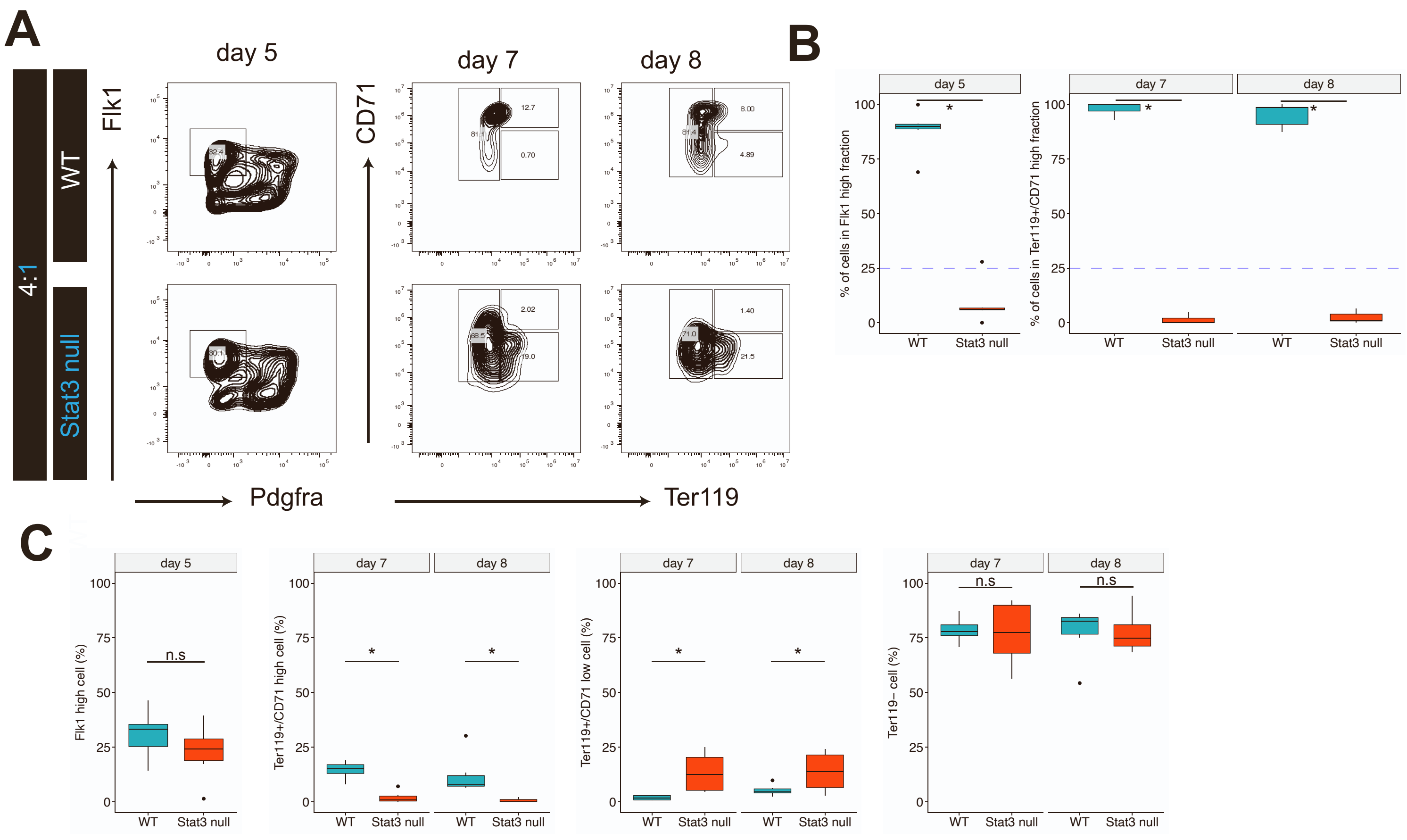

Supplementary Figure 9. Primitive erythroid differentiation in chimeric EBs.

(A) Representative flow cytometry analysis of Flk1/Pdgfra expression in day 5 and Ter119/CD71 expression in day 7 and day 8 in WT and *Stat3* null cells in chimeric EBs, mixing WT and *Stat3* null ESCs at 4:1. (B) Percentages of WT and *Stat3* null cells in the $Flk1^{hi}$ fraction at day 5, and in the $Ter119^{+}/CD71^{hi}$ fraction at day7 and day 8. * $p < 0.05$. (C) Percentages of $Flk1^{hi}$ cells at day 5, and $Ter119^{+}/CD71^{hi}$, $Ter119^{+}/CD71^{low}$, and $Ter119^{-}$ cells at day 7 and day 8 in WT and *Stat3* null cells in chimeric EBs, gated in (A). n.s, not significant. * $p < 0.05$.

Supplementary Table 1. Genotypes of embryos from Stat3+/− intercross

	+/+ or +/-	-/-	Empty decidua
**E3.5**	50	16	
**E4.5**	76	21	
**E5.5**	22	6	
**E6.5**	63	18	2
**E7.5**	45	10	4
**E8.5**	35	5	4
**E9.5**	42	2	5
**E10.5**	18	1	3
**E11.5**	29	4	3

Supplementary Table 2. EpiSC derivation from epiblast of Stat3+/- intercross

	Lines established			
Embryo stage	+/+ or +/-	-/-	Number of epiblast plated	Efficiency
**E6.5**	14	3	17	100%
**E7.5**	10	3	13	100%

Supplementary Table 3. List of genotyping primers

name	Sequence
**STAT3_1**	TTGCTGCTCTCGCTGAAGCGCAGTAGG
**STAT3_2**	CCTGTCTGCCGAATATCATGGTGGAAA
**STAT3_3**	GAGCTGCCTGAGGATAGAGGAACCTGA
